# Genomic prediction using machine learning: a comparison of the performance of regularized regression, ensemble, instance-based and deep learning methods on synthetic and empirical data

**DOI:** 10.1186/s12864-023-09933-x

**Published:** 2024-02-07

**Authors:** Vanda M. Lourenço, Joseph O. Ogutu, Rui A.P. Rodrigues, Alexandra Posekany, Hans-Peter Piepho

**Affiliations:** 1 Center for Mathematics and Applications (NOVA Math) and Department of Mathematics, NOVA SST, 2829-516 Caparica, Portugal; 2 Institute of Crop Science, Biostatistics Unit, University of Hohenheim, Fruwirthstrasse 23, 70599 Stuttgart, Germany; 3 Research Unit of Computational Statistics, Vienna University of Technology, Wiedner Hauptstr. 8-10, 1040 Vienna, Austria

**Keywords:** Genomic prediction, Genomic selection, Breeding value, Predictive accuracy, Predictive ability, High-dimensional data, Supervised machine learning methods

## Abstract

**Background:**

The accurate prediction of genomic breeding values is central to genomic selection in both plant and animal breeding studies. Genomic prediction involves the use of thousands of molecular markers spanning the entire genome and therefore requires methods able to efficiently handle high dimensional data. Not surprisingly, machine learning methods are becoming widely advocated for and used in genomic prediction studies. These methods encompass different groups of supervised and unsupervised learning methods. Although several studies have compared the predictive performances of individual methods, studies comparing the predictive performance of different groups of methods are rare. However, such studies are crucial for identifying (i) groups of methods with superior genomic predictive performance and assessing (ii) the merits and demerits of such groups of methods relative to each other and to the established classical methods. Here, we comparatively evaluate the genomic predictive performance and informally assess the computational cost of several groups of supervised machine learning methods, specifically, *regularized regression* methods, *deep*, *ensemble* and *instance-based* learning algorithms, using one simulated animal breeding dataset and three empirical maize breeding datasets obtained from a commercial breeding program.

**Results:**

Our results show that the relative predictive performance and computational expense of the groups of machine learning methods depend upon both the data and target traits and that for classical regularized methods, increasing model complexity can incur huge computational costs but does not necessarily always improve predictive accuracy. Thus, despite their greater complexity and computational burden, neither the adaptive nor the group regularized methods clearly improved upon the results of their simple regularized counterparts. This rules out selection of one procedure among machine learning methods for routine use in genomic prediction. The results also show that, because of their competitive predictive performance, computational efficiency, simplicity and therefore relatively few tuning parameters, the classical linear mixed model and regularized regression methods are likely to remain strong contenders for genomic prediction.

**Conclusions:**

The dependence of predictive performance and computational burden on target datasets and traits call for increasing investments in enhancing the computational efficiency of machine learning algorithms and computing resources.

**Supplementary Information:**

The online version contains supplementary material available at 10.1186/s12864-023-09933-x.

## Background

Rapid advances in genotyping and phenotyping technologies have enabled widespread and growing use of genomic prediction (GP). The very high dimensional nature of both genotypic and phenotypic data, however, is increasingly limiting the utility of the classical statistical methods. As a result, machine learning (ML) methods able to efficiently handle high dimensional data are becoming widely used in GP. This is especially so because, compared to many other methods used in GP, ML methods possess the significant advantage of being able to model nonlinear relationships between the response and the predictors and complex interactions among predictor variables. However, this often comes at the price of a very high computational burden. Often, however, computational cost is less likely to present serious challenges if the number of SNPs in a dataset is relatively modest but it can become increasingly debilitating as the number of markers grows to millions or even tens of millions. Future advances in computational efficiencies of machine learning algorithms or using high-performance or more efficient programming languages may progressively ameliorate this limitation. Given their growing utility and popularity, it is important to establish the relative predictive performance of different groups of ML methods in GP. Even so, the formal comparative evaluation of the predictive performance of groups of ML methods has attracted relatively little attention. The rising importance of ML methods in plant and animal breeding research and practice, increases both the urgency and importance of evaluating the relative predictive performance of groups of ML methods relative to each other and to classical methods. This can facilitate identification of groups of ML methods that balance high predictive accuracy with low computational cost for routine use with high dimensional phenotypic and genomic data, such as for GP, say.

ML is perhaps one of the most widely used branches of contemporary artificial intelligence. Using ML methods facilitates automation of model building, learning and efficient and accurate predictions. ML algorithms can be subdivided into two major classes: supervised and unsupervised learning algorithms. Supervised regression ML methods encompass regularized regression methods, deep, ensemble and instance-based learning algorithms. Supervised ML methods have been successfully used to predict genomic breeding values for unphenotyped genotypes, a crucial step in genome-enabled selection [1–9]. Furthermore, several studies have assessed the relative predictive performance of supervised ML methods in GP, including two ensemble methods and one instance-based method [5]; four regularized and two adaptive regularized methods [6]; three regularized and five regularized group methods [9] and several deep learning methods [1–4, 8]. However, no study has comprehensively evaluated the comparative predictive performance of all these groups of methods relative to each other or to the classical regularized regression methods. We therefore rigorously evaluate the comparative predictive performance as well as the computational complexity or cost of three groups of popular and state-of-the-art ML methods for GP using one simulated animal dataset and three empirical datasets obtained from a commercial maize breeding program. We additionally offer brief overviews of the mathematical properties of the methods with emphasis on their salient properties, strengths and weaknesses and relationships with each other and with the classical regularization methods. While we offer a somewhat comprehensive review of genomic prediction methods with a specific emphasis on ML, our contribution extends to showcasing novel findings derived from comparative assessments of ML techniques across both real and simulated datasets.

Besides ML methods, Bayesian methods are also becoming widely used for genomic prediction [3, 8, 10]. So, even though our goal is not to provide an exhaustive review of all genomic prediction methods, we offer two Bayesian methods for benchmarking the performance of the ML methods.

The rest of the paper is organized as follows. First we present the synthetic and real datasets. Second, we detail the methods compared in this study. Next, the results from the comparative analyses of the data are presented. Finally, a discussion of the results and closing remarks follow.

## Data

### Simulated (animal) data

We consider one simulated dataset [9], an animal breeding outbred population simulated for the 16-th QTLMAS Workshop 2012 (Additional file 1). The simulation models used to generate the data are described in detail in [11] and are therefore not reproduced here. The dataset consists of 4020 individuals genotyped for 9969 SNP markers. Out of these, 3000 individuals were phenotyped for three quantitative milk traits and the remaining 1020 were not phenotyped (see [9] for details). The goal of the analysis of the simulated dataset is to predict the genomic breeding values (PGBVs) for the 1020 unphenotyped individuals using the available genomic information. The simulated dataset also provides true genomic breeding values (TGBVs) for the 1020 genotypes for all the traits.

As in [9], to enable model fitting for the grouping methods, markers were grouped by assigning consecutive SNP markers systematically to groups of sizes 10, 20, ..., 100 separately for each of the five chromosomes. Typically, the last group of each grouping scheme has fewer SNPs than the prescribed group size. Table 1 summarizes the simulated phenotypic data and highlights differences in the magnitudes of the three simulated quantitative traits $T_1$, $T_2$ and $T_3$.

**Table 1:** Summary statistics for the three quantitative traits ($T_1$, $T_2$ and $T_3$) in the simulated training dataset ($n=3000$ genotypes)

Trait	Min.	1st Qu.	Median	Mean	3rd Qu.	Max.	Sd
$T_1$	-584.993650	-116.244762	-1.711490	-0.000004	112.248515	587.189720	176.518911
$T_2$	-32.233530	-6.502070	0.075480	-0.000004	6.615977	32.514590	9.514060
$T_3$	-0.095720	-0.015893	0.000650	0.000005	0.016450	0.085240	0.024474

### Real (plant) data

For the application to empirical data sets, we use three empirical maize breeding datasets produced by KWS (breeding company) for the Synbreed project during 2010, 2011 and 2012. We first performed separate phenotypic analyses of yield for each of the three real maize data sets to derive the adjusted means used in genomic prediction using a single stage mixed model assuming that genotypes are uncorrelated (Additional file 4, S1 Text). The fixed effect in the mixed model comprised a tester (Tester) with two levels, genotypic group (GRP) with three levels, Tester$\times$GRP and Tester$\times$GRP$\times$G (G=genotype). The random factors were location (LOC), trial (TRIAL) nested within location, replicate (REP) nested within trial and block (BLOCK) nested within replicate. The fitted random effects were LOC, LOC$\times$TRIAL, LOC$\times$TRIAL$\times$REP, LOC$\times$TRIAL$\times$REP$\times$BLOCK, Tester$\times$GRP$\times$SWITCH2$\times$G1 and Tester$\times$GRP$\times$SWITCH1$\times$G2. SWITCH1 and SWITCH2 in the last two effects are operators defined and explained briefly in the supplementary materials (Additional file 4, S1 text; and Additional file 5, Section 1) and in greater detail in [12, 13]. All the three maize datasets involved two testers and three genotypic groups. Accordingly, prior to genomic prediction, we accounted for and removed the effect of the tester$\times$genotypic group (GRP) effect from the adjusted means (lsmeans) of maize yield (dt/ha) by computing the arithmetic mean of the lsmeans for the interaction of testers with GRP for the genotyped lines. This mean was then subtracted from the lsmeans for each tester$\times$GRP interaction term. The resulting deviations were subtracted from the lsmeans of the individual genotypes corresponding to each Tester$\times$GRP interaction. This enabled us not to consider the Tester$\times$GRP effect in the genomic prediction model.

For all the years, every line was genotyped for 32217 SNP markers. A subset of the SNP markers with non-zero variances were split into groups of sizes 10, 20, 30, 40, 50, 60, 70, 80, 90 and 100. Groups were defined by systematically grouping consecutive and spatially adjacent markers, separately for each of 10 chromosomes (Additional file 4, S2 Text). All the checks (standard varieties) and check markers were deleted prior to genomic prediction. More details specific to the three datasets follow (Table 2 summarizes the number of genotypes in the training and validation datasets). The true breeding values are not known in this case.

**Table 2:** Number of genotypes in the training dataset (folds F1-F4) and validation dataset (fold F5) for each of the 10 replicates of the 5-fold cross-validation sets for the 2010, 2011 and 2012 KWS real maize datasets. Individuals were genotyped for a total of 32217 SNPs in all years

	Folds					
	2010		2011		2012	
	F1-F4	F5	F1-F4	F5	F1-F4	F5
**Data**						
Training	859	856	685	688	1104	1108
Validation	214	217	172	169	277	273
**Total**	1073		857		1381	

For each of the 2010, 2011 and 2012 datasets, the genotypes or test crosses were genotyped for 32217 SNPs and randomly split into 5 parts (folds) for 5-fold cross-validation (Additional file 4, S3 Text & S4 Text). The random splitting procedure was repeated 10 times to yield 10 replicates per dataset. The total number of genotypes and the number of individuals assigned to the training and validation sets for each dataset are provided in Table 2.

Table 3 summarizes the KWS phenotypic data for 2010, 2011 and 2012. Each data split for each year (2010, 2011 and 2012) contained approximately 20% of the phenotypic observations and was obtained using stratified random sampling using the algorithm of [14]. The strata were defined by the combinations of the two testers and three genotypic groups.

**Table 3:** Summary statistics for maize yield (dt/ha) in the KWS real maize datasets for 2010, 2011 and 2012

Dataset	Min.	1st Qu.	Median	Mean	3rd Qu.	Max.	Sd
2010	86.597600	121.550000	127.880000	126.806883	132.670000	149.930000	8.297735
2011	101.670000	139.310000	144.710000	144.221949	150.070000	164.060000	8.155595
2012	114.840000	139.160000	143.810000	143.719182	148.470000	169.160000	7.318531

## Methods

In this section we describe the four supervised ML groups of methods.

### Regularized regression methods

Consider the general linear regression model

1 $$\begin{aligned} y_i=\beta _0+\sum \limits _{j=1}^p\beta _jx_{ij}+\varepsilon _i, \ \ i=1,...,n \end{aligned}$$

where $y_i$ is the *i*-th observation of the response variable, $x_{ij}$ is the *i*-th observation of the *j*-th covariate (*p* is the number of all covariates), $\beta _j$ are the regression coefficients (unknown fixed parameters), $\varepsilon _i$ are i.i.d. random error terms with $E(\varepsilon _i)=0$ and $var(\varepsilon _i)=\sigma ^2_e$, where $\sigma ^2_e$ is an unknown random variance, and *n* is the sample size. The ordinary least squares estimator of $\varvec{\beta }=(\beta _0,\dots ,\beta _p)'$, which is unbiased, is obtained by minimizing the residual sum of squares (RSS), i.e.,

$$\begin{aligned} \widehat{\varvec{\beta }}_{ols}=\underset{\varvec{\beta }}{\textit{argmin}} \ \sum \limits _{i=1}^n\Big (y_i-\beta _0-\sum \limits _{j=1}^p \beta _jx_{ij}\Big )^2=\underset{\varvec{\beta }}{\textit{argmin}} \ \Vert {\textbf{y}-\textbf{X}\varvec{\beta }}\Vert _2^2, \end{aligned}$$

where

$$\begin{aligned} \textbf{y}=(y_1\dots ,y_n)', \quad \textbf{X} = \left[ \begin{array}{cccccc} 1 &{} x_{11} &{} x_{12} &{} x_{13} &{} \dots &{} x_{1p} \\ 1 &{} x_{21} &{} x_{22} &{} x_{23} &{} \dots &{} x_{2p} \\ \vdots &{} \vdots &{} \vdots &{} \ddots &{} \vdots \\ 1 &{} x_{n1} &{} x_{n2} &{} x_{n3} &{} \dots &{} x_{np} \end{array}\right] \quad \text {and}\ \Vert . \Vert _2 \text { is the}\ \ell _{2}\text {-norm.} \end{aligned}$$

This estimator is typically not suitable when the design matrix $\textbf{X}$ is less than full rank ($\textbf{X}$ has a full rank if the number of its linearly independent rows or columns $k=\min (p,n)$) or is close to collinearity (i.e., the covariates are close to being linear combinations of one another) [15]; problems that are frequently associated with $p>>n$.

In genomic prediction (GP) one is interested in estimating the *p* regression coefficients $\beta _j$ so that genomic breeding values of non-phenotyped genotypes can be predicted from the fitted model. The response variable $\textbf{y}$ is often some quantitative trait and the $\beta _j$’s are the coefficients of molecular markers spanning the whole genome, usually Single Nucleotide Polymorphisms (SNPs). Because in GP typically $p>>n$, the ordinary least squares (OLS) estimator breaks down and thus other methods for estimating $\varvec{\beta }$ in (1) must be sought. Indeed, the increasingly high dimensional nature of high-throughput SNP-marker datasets has prompted increasing use of the power and versatility of regularization methods in genomic prediction to simultaneously select and estimate important markers and account for multicollinearity [5, 6].

Without loss of generality, we assume, consistent with the standard practice in regularized estimation where a distance-based metric is used for prediction, that the response variable is mean-centered whereas the covariates in (1) are standardized, so that

$$\begin{aligned} \sum \limits _{i=1}^ny_i=0,\qquad \sum \limits _{i=1}^nx_{ij}=0\quad \text {and} \quad n^{-1}\sum \limits _{i=1}^nx_{ij}^2=1,\quad j=1,\dots ,p. \end{aligned}$$

Regularized regression methods minimize a non-negative loss function (RSS or other) plus a non-negative penalty function. Standardizing the covariates prior to model fitting ensures that the penalty is applied evenly to all covariates. Mean-centering the response and the covariates is usually done for notational simplicity but also eliminates the need to estimate the intercept $\beta _0$.

After the penalized models have been fit, the final estimates are obtained by back transformation to the original scale by re-introducing an intercept ($\beta _0$). In particular, for a mean-centered response $\textbf{y}$ and standardized predictor $\textbf{X}^{\varvec{*}}$, predictions are obtained by

$$\begin{aligned} \widehat{\textbf{y}}=\beta _0+\sum \limits _{j=1}^p{\textbf{X}}^*_j\widehat{\beta }^*_j \end{aligned}$$

with $\widehat{\varvec{\beta }}^*=(\widehat{\beta }^*_1,\dots ,\widehat{\beta }^*_p)$, the regression coefficients from the model fit with the mean-centered response $\textbf{y}$ and standardized covariates $\textbf{X}^{\varvec{*}}$, ${\textbf{X}}^*_j=(x_{1j},\dots ,x_{nj})'$ the *j*-th covariate and $\beta _0=\bar{\textbf{y}}$. One can also choose to predict using the original predictor $\textbf{X}^{\varvec{*}}$ without standardization. In that case one should back transform the $\widehat{\beta }^*_j$ to the original scale and consider

$$\begin{aligned} \widehat{\textbf{y}}=\beta _0+\sum \limits _{j=1}^p{\textbf{X}}^*_j\widehat{\beta }_j \end{aligned}$$

with $\widehat{\beta }_j=\widehat{\beta }^*_j/s_j$, $s_j=\sqrt{n^{-1}\sum \limits _{i=1}^nx_{ij}^2}$ the standard deviation of the j-th covariate ${\textbf{X}}^*_j$ and $\beta _0 = \bar{\textbf{y}}- {\tilde{\textbf{X}}} \widehat{\varvec{\beta }}$, where ${\tilde{\textbf{X}}}_j=(m_j,\dots ,m_j)'$ is a vector of size *n* with $m_j$ being the mean of the *j*-th covariate ${\textbf{X}}^*_j$.

The primary goal of regularization methods is to reduce model complexity resulting from high dimensionality by reducing the number of predictors in the model. This is achieved by either shrinking some coefficients to become exactly zero, and so drop out of the model, or shrinking all coefficients to be close to zero and each other but not exactly zero. Ideally, a desirable estimator of $\varvec{\beta }$ should (i) correctly select the nonzero coefficients with probability converging to 1 (i.e. with near certainty; *selection consistency*) and (ii) yield estimators of the nonzero coefficients that are asymptotically normal with the same means and covariances that they would have if the zero coefficients were known exactly in advance (*asymptotic normality*). An estimator satisfying these two conditions is said to possess the *oracle* property [16, 17].

For the remainder of the paper, we assume that ${\textbf {X}}$ is a $n\times p$ marker matrix (e.g., with the genotypes $\{aa,Aa,AA\}$ coded as $\{0,1,2\}$ or $\{-1,0,1\}$ for *p* biallelic SNPs under an additive model) with ${\textbf {X}}_j$ denoting the *j*-th SNP covariate and $\varvec{\beta }=(\beta _1,\dots ,\beta _p)$ denoting the unknown vector of marker effects. Table 4 (upper half) summarizes the methods discussed in this sub-section.

**Table 4:** A summary of the estimators and penalty functions for the bridge-type and adaptive bridge-type regularized regression methods used in this study. The adaptive methods have an *a* prefix in their names

Method	Penalty	Estimator	
**bridge**	$p_{\lambda ,\gamma }(\varvec{\beta })=\lambda \sum \limits _{j=1}^p|\beta _j|^{\gamma }$	$\widehat{\varvec{\beta }}_{bridge}=\underset{\varvec{\beta }}{\textit{argmin}} \ \Big \{\text {RSS} + \lambda \sum \limits _{j=1}^p|\beta _j|^{\gamma } \Big \}, \ \gamma >0, \ \lambda \ge 0$	(2)
$\bullet \ \gamma =1$:			
LASSO	$p_{\lambda }(\varvec{\beta })=\lambda \Vert \varvec{\beta }\Vert _1$	$\widehat{\varvec{\beta }}_{lasso}=\underset{\varvec{\beta }}{\textit{argmin}} \ \Big \{\text {RSS} + \lambda \Vert \varvec{\beta }\Vert _1\Big \}$	(3)
$\bullet \ \gamma =2$:			
ridge	$p_{\lambda }(\varvec{\beta })=\lambda \Vert \varvec{\beta }\Vert _2^2$	$\widehat{\varvec{\beta }}_{ridge}=\underset{\varvec{\beta }}{\textit{argmin}} \ \Big \{\text {RSS} + \lambda \Vert \varvec{\beta }\Vert _2^2\Big \}$	(4)
$\bullet$ Combination of LASSO and ridge penalties ($\gamma =1,2$, respectively):			
ENET	$p_{\varvec{\lambda }}(\varvec{\beta })=\lambda _1\Vert \varvec{\beta }\Vert _1+\lambda _2\Vert \varvec{\beta }\Vert _2^2$	$\widehat{\varvec{\beta }}_{enet}=(1+\lambda _2)\times \underset{\varvec{\beta }}{\textit{argmin}}\Big \{ \text {RSS} + \lambda _1\Vert \varvec{\beta }\Vert _1+ \lambda _2 \Vert \varvec{\beta }\Vert _2^2\Big \}$	(6)
a**bridge**	$p_{\lambda ,\gamma }(\varvec{\beta })=\lambda \sum \limits _{j=1}^p w_j|\beta _j|^{\gamma }$	$\widehat{\varvec{\beta }}_{\texttt {a}bridge}=\underset{\varvec{\beta }}{\textit{argmin}} \ \Big \{\text {RSS} + \lambda \sum \limits _{j=1}^p{w}_j|\beta _j|^{\gamma }\Big \}$	(7)
$\bullet \ \gamma =1$:			
aLASSO	$p_{\lambda }(\varvec{\beta })=\lambda \Vert {\textbf {w}}\varvec{\beta }\Vert _1$	$\widehat{\varvec{\beta }}_{\texttt {a}lasso}=\underset{\varvec{\beta }}{argmin} \ \Big \{\text {RSS} + \lambda \Vert {\textbf {w}}\varvec{\beta }\Vert _1\Big \}$	(8)
$\bullet$ Combination of aLASSO and ridge penalties ($\gamma =1,2$, respectively):			
aENET	$p_{\varvec{\lambda }}(\varvec{\beta }) = \lambda _1\Vert {\textbf {w}}\varvec{\beta }\Vert _1+ \lambda _2 \Vert \varvec{\beta }\Vert _2^2$	$\widehat{\varvec{\beta }}_{\texttt {a}enet}= k\times \underset{\varvec{\beta }}{argmin}\ \ \Big \{\text {RSS} + \lambda _1\Vert {\textbf {w}}\varvec{\beta }\Vert _1+ \lambda _2 \Vert \varvec{\beta }\Vert _2^2\Big \}$	(9)

#### Bridge-type estimators

The most popular regularization methods in genomic prediction include ridge regression (RR; [18]), the least absolute shrinkage and selection operator (LASSO; [19]) and the elastic net (ENET; [20]). All these methods are special cases of the bridge estimator [15, 21] given by

2 $$\begin{aligned} \widehat{\varvec{\beta }}_{bridge}=\underset{\varvec{\beta }}{\textit{argmin}} \ \Big \{\text {RSS} + \lambda \sum \limits _{j=1}^p|\beta _j|^{\gamma }\Big \}, \quad \gamma >0, \quad \lambda \ge 0, \end{aligned}$$

where the *regularization* parameter $\lambda$ balances the goodness-of-fit against model complexity and the *shrinkage* parameter $\gamma$ determines the order of the penalty function. The optimal combination of $\lambda$ and $\gamma$ can be selected adaptively for each dataset by grid search using cross-validation (CV; if the focus is on predictive performance) or by information criteria (e.g., AIC or BIC; if the focus is on model fit). Bridge regression automatically selects relevant predictors when $0<\gamma \le 1$, shrinks the coefficients when $\gamma >1$ and reduces to subset selection when $\gamma =0$. The bridge estimator reduces to the LASSO estimator when $\gamma =1$ and to the ridge estimator when $\gamma =2$. Specifically,

3 $$\begin{aligned} \widehat{\varvec{\beta }}_{lasso}=\underset{\varvec{\beta }}{\textit{argmin}} \ \Big \{\text {RSS} + \lambda \Vert \varvec{\beta }\Vert _1\Big \}, \end{aligned}$$

where $\Vert . \Vert _1$ is the $\ell _1$-norm, and

4 $$\begin{aligned} \widehat{\varvec{\beta }}_{ridge}=\underset{\varvec{\beta }}{\textit{argmin}} \ \Big \{\text {RSS} + \lambda \Vert \varvec{\beta }\Vert _2^2\Big \}. \end{aligned}$$

The bridge estimator also enjoys several other useful and interesting properties (see [22, 23] for more details). We summarize these salient properties with emphasis on the special cases of the LASSO ($\gamma =1$) and the ridge estimators ($\gamma =2$).

1. The asymptotic properties of bridge estimators have been studied in detail by [22]. In particular, where $p<n$, with *p* increasing to infinity as *n* grows, and under appropriate regularity conditions, bridge estimators enjoy the oracle property for $0<\gamma <1$. This implies that neither the LASSO nor the ridge estimator possesses the oracle property [16, 17]. If $p>>n$ and no assumptions are imposed on the covariate matrix, then the regression parameters are generally non-identifiable. However, if a suitable structure is assumed for the covariate matrix, then bridge estimators achieve consistent variable selection and estimation [22].
2. Although the LASSO estimator performs automatic variable selection, it is a biased and inconsistent estimator [24, 25]. Moreover, it is unstable with high-dimensional data because it
  (i) cannot select a larger number of predictors *p* than the sample size *n* if $p>>n$;
  (ii) arbitrarily selects one member of a set of pairwise highly correlated predictors and ignores the other.
3. The ridge estimator performs well for many predictors each of which has a small effect but cannot shrink the coefficients to become exactly zero. Moreover, the ridge estimator
  (i) prevents coefficients of linear regression models with many correlated variables from being poorly determined and exhibiting high variance;
  (ii) shrinks coefficients of correlated predictors equally towards zero and towards each other;
  (iii) retains all predictor variables in the model leading to complex and less interpretable models.
  In addition, RR has close connections with marker-based best linear unbiased prediction (BLUP) and genomic best linear unbiased prediction (GBLUP) [26], which we clarify in what follows. The ridge estimator is given by
  $$\begin{aligned} \widehat{\varvec{\beta }}_{ridge}= ({\textbf {X}}'{} {\textbf {X}}+\lambda {\textbf {I}})^{-1}{} {\textbf {X}}'{} {\textbf {y}}, \end{aligned}$$
  where, if $\lambda$ is estimated by cross-validation as suggested above, then the ridge estimator may be denoted by RR-CV. Another way of looking at the ridge estimator is to assume in (1) that $\varvec{\beta }\sim N({\textbf {0}},{\textbf {I}}\sigma ^2_{\beta })$ is a random vector of unknown marker effects and that $\varvec{\varepsilon }\sim N({\textbf {0}},{\textbf {I}}\sigma ^2_{e})$ is an unknown random error term, where $\sigma ^2_{\beta }$ and $\sigma ^2_{e}$ are the unknown marker-effect and error variances, respectively. Model (1), written in matrix form as
  5 $$\begin{aligned} {\textbf{y}}={\textbf{X}}\varvec{\beta }+\varvec{\varepsilon }, \end{aligned}$$
  is now a linear mixed model and hence, the variances can be estimated via the restricted maximum likelihood (REML) method. Observing that ${\textbf{y}}\sim N({\varvec{0}},{\textbf{K}}\sigma ^2_{\beta }+{\textbf{I}}\sigma ^2_{\varepsilon })$, where ${\textbf{K}}={\textbf {X}}'{} {\textbf {X}}$ is the kinship or genomic relationship matrix, the BLUP solution for the marker effects under model (5) is given by ([27]; p.270)
  $$\begin{aligned} \widehat{\varvec{\beta }}_{BLUP}=\text {cov}(\varvec{\beta },{\textbf {y}})\times (\text {var}({\textbf {y}}))^{-1}{} {\textbf {y}}={\textbf {X}}'\sigma ^2_{\beta }({\textbf{K}}\sigma ^2_{\beta }+{\textbf{I}}\sigma ^2_{\varepsilon })^{-1}{\textbf{y}} = {\textbf {X}}'({\textbf {K}}+{\textbf {H}})^{-1}{} {\textbf {y}} \end{aligned}$$
  Now defining ${\textbf {H}}={\textbf {I}} \frac{\sigma ^2_{\varepsilon }}{\sigma ^2_{\beta }}$ to simplify the notation and pre-multiplying $\widehat{\varvec{\beta }}_{BLUP}$ with $({\textbf {X}}'{} {\textbf {X}}+{\textbf {H}})^{-1}{} {\textbf {X}}'({\textbf {K}}+{\textbf {H}}){\textbf {K}}^{-1}{} {\textbf {X}}$ we obtain
  $$\begin{aligned} ({\textbf {X}}'{} {\textbf {X}}+{\textbf {H}})^{-1}{} {\textbf {X}}'({\textbf {K}}+{\textbf {H}}){\textbf {K}}^{-1}{} {\textbf {X}}\widehat{\varvec{\beta }}_{BLUP}=({\textbf {X}}'{} {\textbf {X}}+{\textbf {H}})^{-1}{} {\textbf {X}}'{} {\textbf {y}}. \end{aligned}$$
  Finally, observing that $({\textbf {X}}'{} {\textbf {X}}+{\textbf {H}})^{-1}{} {\textbf {X}}'({\textbf {K}}+{\textbf {H}}){\textbf {K}}^{-1}{} {\textbf {X}}={\textbf {X}}'{} {\textbf {K}}^{-1}{} {\textbf {X}}$ (see Appendix) and that ${\textbf {X}}'{} {\textbf {K}}^{-1}{} {\textbf {X}}{} {\textbf {X}}'={\textbf {X}}'$ we find that
  $$\begin{aligned} \widehat{\varvec{\beta }}_{BLUP}=\Big ({\textbf {X}}'{} {\textbf {X}}+ \frac{\sigma ^2_{e}}{\sigma ^2_{\beta }}{} {\textbf {I}}\Big )^{-1}{} {\textbf {X}}'{} {\textbf {y}}, \end{aligned}$$
  establishing the equivalence of BLUP and RR [28, 29] and that one can actually estimate the ridge parameter $\lambda$ by $\widehat{\lambda }=\frac{\widehat{\sigma }^2_{e}}{\widehat{\sigma }^2_{\beta }}$. Because we use REML to estimate the two variance components in $\widehat{\varvec{\beta }}_{BLUP}$, we refer to this RR appproach as RR-REML. Our basic regression model (5) can be written as
  $$\begin{aligned} {\textbf {y}} = {\textbf {g}} + \varvec{\varepsilon }, \end{aligned}$$
  where, ${\textbf {g}}={\textbf {X}}\varvec{\beta }$. Making the same assumptions as for RR-REML, i.e., assuming that $\varvec{\beta }\sim N({\textbf {0}},{\textbf {I}}\sigma ^2_{\beta })$ and $\varvec{\varepsilon }\sim N({\textbf {0}},{\textbf {I}}\sigma ^2_{e})$, we have that ${\textbf {g}}\sim N({\textbf {0}},{\textbf {K}}\sigma ^2_{\beta })$. The BLUP of ${\textbf {g}}$, also known as genomic estimated breeding values (GEBV) or gBLUP, under this model is ([27]; p.270)
  $$\begin{aligned} \widehat{{\textbf {g}}}_{BLUP}=\text {cov}({\textbf {g}},{\textbf {y}})\times (\text {var}({\textbf {y}}))^{-1}{} {\textbf {y}} = {\textbf {K}}\sigma ^2_{\beta }({\textbf {K}}\sigma ^2_{\beta }+{\textbf {I}}\sigma ^2_{\varepsilon })^{-1}{} {\textbf {y}} = {\textbf {K}}\Big ({\textbf {K}}+{\textbf {I}} \frac{\sigma ^2_{\varepsilon }}{\sigma ^2_{\beta }}\Big )^{-1}{} {\textbf {y}}. \end{aligned}$$
  Now pre-multiplying $\widehat{{\textbf {g}}}_{BLUP}$ with ${\textbf {X}}({\textbf {X}}'{} {\textbf {X}}+{\textbf {H}})^{-1}{} {\textbf {X}}'({\textbf {K}}+{\textbf {H}}){\textbf {K}}^{-1}$ we obtain
  $$\begin{aligned} {\textbf {X}}({\textbf {X}}'{} {\textbf {X}}+{\textbf {H}})^{-1}{} {\textbf {X}}'({\textbf {K}}+{\textbf {H}}){\textbf {K}}^{-1}\widehat{{\textbf {g}}}_{BLUP}={\textbf {X}}({\textbf {X}}'{} {\textbf {X}}+{\textbf {H}})^{-1}{} {\textbf {X}}'{} {\textbf {y}}={\textbf {X}}\widehat{\varvec{\beta }}_{BLUP}. \end{aligned}$$
  Finally, observing that ${\textbf {X}}({\textbf {X}}'{} {\textbf {X}}+{\textbf {H}})^{-1}{} {\textbf {X}}'({\textbf {K}}+{\textbf {H}}){\textbf {K}}^{-1}={\textbf {I}}$ (see Appendix), we find that $\widehat{{\textbf {g}}}_{BLUP}={\textbf {X}}\widehat{\varvec{\beta }}_{BLUP}$ establishing the equivalence of RR-REML and gBLUP [30, 31].
4. Due to the nature of the $\ell _1$ penalty, particularly for high values of $\lambda$, the LASSO estimator will shrink many coefficients to exactly zero, something that never happens with the ridge estimator.

#### Elastic net estimator

The elastic net estimator blends two bridge-type estimators, the LASSO and the ridge, to produce a composite estimator that reduces to the LASSO when $\lambda _2=0$ and to the ridge when $\lambda _1=0$. Specifically, the elastic net estimator is specified by

6 $$\begin{aligned} \widehat{\varvec{\beta }}_{enet}=k\times \underset{\varvec{\beta }}{\textit{argmin}} \Big \{\text {RSS} + \lambda _1\Vert \varvec{\beta }\Vert _1+ \lambda _2 \Vert \varvec{\beta }\Vert _2^2\Big \}. \end{aligned}$$

with $k=1+\lambda _2$ if the predictors are standardized (as we assume) or $k=1+\lambda _2/n$ otherwise. Even when $\lambda _1,\lambda _2\ne 0$, the elastic net estimator behaves much like the LASSO but with the added advantage of being robust to extreme correlations among predictors. Moreover, the elastic net estimator is able to select more than *n* predictors when $p>>n$. Model sparsity occurs as a consequence of the $\ell _1$ penalty term. Mazumder et al. [32] proposed an estimation procedure based on sparse principal components analysis (PCA), which produces an even more sparse model than the original formulation of the elastic net estimator [20]. Because it blends two bridge-type estimators, neither of which enjoys the oracle property, the ENET also lacks the oracle property.

Other competitive regularization methods that are asymptotically oracle efficient ($p<n$ with *p* increasing to infinity with *n*), which do not fall into the category of bridge-type estimators, are the *smoothly clipped absolute deviations* (SCAD [17, 33]) and the *minimax concave penalty* (MCP [25, 34]) methods. Details of the penalty functions and other important properties of both methods can be found elsewhere [9, 35].

### Adaptive regularized regression methods

The adaptive regularization methods are extensions of the regularized regression methods that allow the resulting estimators to achieve the oracle property under certain regularity conditions. Table 4 (lower half) summarizes the adaptive methods considered here.

#### Adaptive bridge-type estimators

Adaptive bridge estimators extend the bridge estimators by introducing weights in the penalty term. More precisely,

7 $$\begin{aligned} \widehat{\varvec{\beta }}_{\texttt {a}bridge}=\underset{\varvec{\beta }}{\textit{argmin}} \ \Big \{\text {RSS} + \lambda \sum \limits _{j=1}^p{w}_j|\beta _j|^{\gamma }\Big \}, \quad \gamma >0, \quad \lambda \ge 0 \end{aligned}$$

where $\{{w}_j\}_{j=1}^p$ are adaptive data-driven weights. As with the bridge-type estimator, the adaptive bridge estimator simplifies to the adaptive LASSO (aLASSO) estimator when $\gamma =1$ and to the adaptive ridge estimator when $\gamma =2$. Chen et al. [36] studied the properties of adaptive bridge estimators for the particular case when $p<n$ (with *p* increasing to infinity with *n*), $0<\gamma <2$ and ${w}_j=(\vert \widehat{\beta }_j^{init}\vert )^{-1}$ with $\widehat{\varvec{\beta }}^{init}=\widehat{\varvec{\beta }}_{ols}$. They showed that for $0<\gamma <1$, and under additional model assumptions, adaptive bridge estimators enjoy the oracle property. For $p>>n$, $\widehat{\varvec{\beta }}_{ols}$ cannot be computed and thus other initial estimates, such as $\widehat{\varvec{\beta }}_{ridge}$, have to be used. Theoretical properties of the adaptive bridge estimator for $p>>n$ do not seem to have been well studied thus far.

The adaptive LASSO estimator was proposed by [37] to remedy the problem of the lack of the oracle property of the LASSO estimator [16, 17]. The penalty for the adaptive LASSO is given by (adaptive bridge estimator with $\gamma =1$)

$$\begin{aligned} \texttt{p}_{\lambda }(\varvec{\beta })=\lambda \sum \limits _{j=1}^p{w}_j|\beta _j| \end{aligned}$$

where the adaptive data-driven weights $\{{w}_j\}_{j=1}^p$ can be computed as ${w}_j=(\vert \widehat{\beta }_j^{init}\vert )^{-\nu }$ with $\widehat{\varvec{\beta }}^{init}$ an initial root-*n* consistent estimate of $\varvec{\beta }$ obtained through least squares (or ridge regression if multicollinearity is important) and $\nu$ is a positive constant. Consequently,

8 $$\begin{aligned} \widehat{\varvec{\beta }}_{\texttt {a}lasso}=\underset{\varvec{\beta }}{\textit{argmin}} \ \Big \{\text {RSS} + \lambda \Vert {\textbf {w}}\varvec{\beta }\Vert _1\Big \} \end{aligned}$$

with $\nu$ chosen appropriately, performs as well as the oracle, i.e., the adaptive LASSO achieves the oracle property. Nevertheless, this estimator still inherits the LASSO’s instability with high dimensional data. The values of $\lambda$ and $\nu$ can be simultaneously selected from a grid of values, with values of $\nu$ selected from $\{0.5,1,2\}$, using two-dimensional cross-validation [37].

Grandvalet [38] shows that the adaptive ridge estimator (adaptive bridge estimator with $\gamma =2$) is equivalent to the LASSO in the sense that both produce the same estimate and thus the adaptive ridge is not considered further.

#### Adaptive elastic-net

The adaptive elastic-net (aENET) combines the ridge and aLASSO penalties to achieve the oracle property [39] while at the same time alleviating the instability of the aLASSO with high dimensional data. The method first computes $\widehat{\varvec{\beta }}_{enet}$ as described above for the elastic net estimator, then constructs the adaptive weights as $\widehat{w}_j=(|\widehat{\beta }_{j,enet}|)^{-\nu }$, where $\nu$ is a positive constant, and then solves

9 $$\begin{aligned} \widehat{\varvec{\beta }}_{\texttt {a}enet}=k\times \underset{\varvec{\beta }}{\textit{argmin}} \Big \{\text {RSS} + \lambda _1\Vert {\textbf {w}}\varvec{\beta }\Vert _1+ \lambda _2 \Vert \varvec{\beta }\Vert _2^2\Big \}, \end{aligned}$$

where $k=1+\lambda _2$ if the predictors are standardized (as we assume) or $k=1+\lambda _2/n$ otherwise. In particular, when $\lambda _2=0$ the adaptive elastic-net reduces to the aLASSO estimator. This is also the case when the design matrix is orthogonal regardless of the value of $\lambda _2$ [20, 37, 39].

Other adaptive regularization methods are the *multi-step adaptive ENET* (maENET), the *adaptive smoothly clipped absolute deviations* (aSCAD) and the *adaptive minimax concave penalty* (aMCP) methods. Details of the penalty functions and noteworthy properties of the latter three methods are summarized elsewhere [6, 40].

### Regularized group regression methods

Regularized regression methods that select individual predictors do not exploit information on potential grouping structure among markers, such as that arising from the association of markers with particular Quantitative Trait Loci (QTL) on a chromosome or haplotype blocks, to enhance the accuracy of genomic prediction. The nearby SNP markers in such groups are linked, producing highly correlated predictors. If such grouping structure is present but is ignored by using models that select individual predictors only, then such models may be inefficient or even inappropriate, reducing the accuracy of genomic prediction [9]. Regularized group regression methods are regularized regression methods with penalty functions that enable the selection of the important groups of covariates and include group bridge (gbridge), group LASSO (gLASSO), group SCAD (gSCAD) and group MCP (gMCP) methods (see [9, 41–46] for detailed reviews). Some grouping methods such as the group bridge, sparse group LASSO (sgLASSO) and group MCP, besides allowing for group selection, also select the important members of each group [43] and are therefore said to perform bi-level selection, i.e., group-wise and within-group variable selection. Bi-level selection is appropriate if predictors are not distinct but have a common underlying grouping structure.

Estimators and penalty functions for the regularized grouped methods can be formulated as follows. Consider subsets $A_1,\ldots ,A_L$ of $\{1,\dots ,p\}$ (*L* being the total number of covariate groups), representing known covariate groupings of design vectors, which may or may not overlap. Let $\varvec{\beta }_{A_l}=(\beta _k , k \in A_l)$ be the regression coefficients in the *l*-th group and $p_l$ the cardinality of the *l*-th group (i.e., the number of unique elements in $A_l$). Regularized group regression methods estimate $\varvec{\beta }=(\varvec{\beta }_{A_1},...,\varvec{\beta }_{A_L})'$ by minimizing

10 $$\begin{aligned} F_{\lambda ,\gamma }^L(\varvec{\beta })= \sum \limits _{i=1}^n\Big (y_i-\sum \limits _{l=1}^L {\textbf{X}}_{il}\varvec{\beta }_{A_l}\Big )^2 + \texttt{p}_{\lambda }(\varvec{\beta }), \end{aligned}$$

where ${\textbf{X}}_{.l}$ is a matrix with columns corresponding to the predictors in group *l*.

Because $\sum \limits _{i=1}^n\Big (y_i-\sum \limits _{l=1}^L {\textbf{X}}_{il}\varvec{\beta }_{A_l}\Big )^2$ in (10) is equivalent to RSS some authors use the RSS formulation directly. It is assumed that all the covariates belong to at least one of the groups. Table 5 summarizes the methods described in this section.

**Table 5:** Penalty functions and estimators for some group regularized regression methods used in this study

Method	Penalty	Estimator	
gbridge	$p_{\lambda ,\gamma }(\varvec{\beta })= \lambda \sum \limits _{l=1}^L c_l\Vert \varvec{\beta }_{A_l}\Vert _1^{\gamma }$	$\widehat{\varvec{\beta }}_{\texttt {g}bridge}=\underset{\varvec{\beta }}{\textit{argmin}} \ \Big \{\text {RSS} + \lambda \sum \limits _{l=1}^L c_l\Vert \varvec{\beta }_{A_l}\Vert _1^{\gamma }\Big \}$	(11)
gLASSO	$\texttt{p}_{\lambda }(\varvec{\beta })=\lambda \sum \limits _{l=1}^L \sqrt{p_l}\Vert \varvec{\beta }_{A_l}\Vert _2$	$\widehat{\varvec{\beta }}_{\texttt {g}lasso}=\underset{\varvec{\beta }}{\textit{argmin}} \ \Big \{\text {RSS} + \lambda \sum \limits _{l=1}^L \sqrt{p_l}\Vert \varvec{\beta }_{A_l}\Vert _2\Big \}$	(12)
sgLASSO	$\texttt{p}_{\lambda ,\alpha }(\varvec{\beta })= \alpha \lambda ||\varvec{\beta }||_1 + (1-\alpha ) \lambda \sum \limits _{l=1}^L \sqrt{g_l}||\varvec{\beta }_l||_2$	$\widehat{\varvec{\beta }}_{\texttt {sg}lasso}\underset{\varvec{\beta }}{\textit{argmin}} \ \Big \{\text {RSS} + \alpha \lambda ||\varvec{\beta }||_1 + (1-\alpha ) \lambda \sum \limits _{l=1}^L \sqrt{g_l}||\varvec{\beta }_l||_2 \Big \}$	(13)

#### Group bridge-type estimators

Group bridge-type estimators use in (10) the penalty term $p_{\lambda }(\varvec{\beta })=\lambda \sum \limits _{l=1}^L c_l\Vert \varvec{\beta }_{A_l}\Vert _1^{\gamma }$ with $c_l$ constants that adjust for the different sizes of the groups. The group bridge-type estimators are thus obtained as

11 $$\begin{aligned} \widehat{\varvec{\beta }}_{\texttt {g}bridge}=\underset{\varvec{\beta }}{\textit{argmin}} \ \text {RSS} + \lambda \sum \limits _{l=1}^L c_l\Vert \varvec{\beta }_{A_l}\Vert _1^{\gamma },\quad \gamma >0,\quad \lambda \ge 0. \end{aligned}$$

A simple and usual choice for the $c_l$ constants consists in considering each $c_l\propto p_l^{1-\gamma }$. When $0<\gamma <1$ group bridge can be used simultaneously for group and individual variable selection. Also, note that under these assumptions, the group bridge estimator correctly selects groups with nonzero coefficients with probability converging to one under reasonable regularity conditions, i.e., it enjoys the *oracle group selection* property (see [47] for details). When the group sizes are all equal to one, i.e., $p_l=1 \ \forall \ 1\le l \le L$, then group bridge estimators reduce to the bridge estimators.

#### Group LASSO and sparse group LASSO

Group LASSO regression uses in (10) the penalty function $\texttt{p}_{\lambda }(\varvec{\beta })=\lambda \sum \limits _{l=1}^L\sqrt{p_l}||\varvec{\beta }_{A_l}||_2$. The group LASSO estimator is thus given by

12 $$\begin{aligned} \widehat{\varvec{\beta }}_{\texttt {g}lasso}=\underset{\varvec{\beta }}{\textit{argmin}} \ \Big \{\text {RSS} + \lambda \sum \limits _{l=1}^L \sqrt{p_l}||\varvec{\beta }_{A_l}||_2\Big \},\quad \lambda \ge 0. \end{aligned}$$

Unlike the group bridge estimator ($0<\gamma <1$), gLASSO is designed for group selection, but does not select individual variables within the groups. Indeed, its formulation is more akin to that of the adaptive ridge estimator [47]. As with the group-bridge estimator, when the group sizes are all equal to one, i.e., $p_l=1 \ \forall \ 1\le l \le L$, the gLASSO estimator reduces to the LASSO estimator.

Because the gLASSO does not yield sparsity within a group (it either discards or retains a whole group of covariates) the sparse group lasso (sgLASSO), which blends the LASSO and the gLASSO penalties, was proposed [48, 49]. Specifically, the sgLASSO estimator is given by

13 $$\begin{aligned} \widehat{\varvec{\beta }}_{\texttt {sg}lasso}= \underset{\varvec{\beta }}{\textit{argmin}} \ \Big \{\text {RSS} + (1-\alpha ) \lambda \sum \limits _{l=1}^L \sqrt{g_l}||\varvec{\beta }_l||_2 + \alpha \lambda ||\varvec{\beta }||_1\Big \}, \end{aligned}$$

where $\alpha \in [0,1]$ provides a convex combination of the lasso and group lasso penalties ($\alpha =0$ gives the gLASSO fit, $\alpha =1$ gives the LASSO fit). The gLASSO is superior to the standard LASSO under the strong group sparsity and certain other conditions, including a group sparse eigenvalue condition [50]. Because the sgLASSO lacks the oracle property, the adaptive sparse group LASSO was recently proposed to remedy this drawback [51].

Note that there are two types of sparsity, i.e., (i) “groupwise sparsity”, which refers to the number of groups with at least one nonzero coefficient, and (ii) “within group sparsity” that refers to the number of nonzero coefficients within each nonzero group. The “overall sparsity” usually refers to the total number of non-zero coefficients regardless of grouping.

Other group regularization methods are the *hierarchical group LASSO* (hLASSO), the *group smoothly clipped absolute deviations* (gSCAD) and the *group minimax concave penalty* (gMCP) methods. Details of the penalty functions and salient properties of these methods can be found in [9, 52–55].

### Bayesian regularized estimators

The two Bayesian methods we consider are based on the Bayesian basic linear regression model [10]. They assume a continuous response ${\textbf{y}}=(y_1, \ldots , y_n)$ so that the regression equation can be represented as $y_i = \eta _i + \varepsilon _i$, where $\eta _i$ is a linear predictor (the expected value of $y_i$ given predictors) and $\varepsilon _i$ are independent normal model residuals with mean zero and variance $w_i^2\sigma ^2_{\varepsilon }$, with $w_i$ representing user defined weights and $\sigma ^2_{\varepsilon }$ is a residual variance parameter. The model structure for the linear predictor $\varvec{\eta }$ is constructed as follows

$$\begin{aligned} \varvec{\eta } = {\varvec{1}} \mu + \sum \limits _{j=1}^{p} {\textbf{X}}_j\varvec{\beta }_j \end{aligned}$$

with an intercept $\mu$ (equivalent to $\beta _0$ in equation (1)), design $n\times p$ matrix ${\textbf{X}}$ for predictor vectors ${\textbf{X}}_j = (x_{ij})$ and fixed effects vectors $\varvec{\beta }_j$ associated with the the predictors ${\textbf{X}}_j$.

The likelihood function of the data has the following conditional distribution:

$$\begin{aligned} p({\textbf{y}}|\varvec{\theta }) = \prod \limits _{i=1}^{n} N\left( y_i|\mu + \sum \limits _{j=1}^{p} x_{ij}\beta _{j}, \sigma _{\varepsilon }^2 w_i^2 \right) \end{aligned}$$

with the general parameter vector $\varvec{\theta }$ representing the vector of all unknowns, such as the intercept, all the regression coefficients and random effects, the residual variance as well as parameters and hyper-parameters subject to inference in the hierarchical Bayesian model.

The prior distribution factorises as follows:

$$\begin{aligned} p(\varvec{\theta }) = p(\mu )p(\sigma ^2_{\varepsilon })\prod \limits _{j=1}^{p}p(\varvec{\beta }_j)). \end{aligned}$$

In the basic form of the model the following prior settings are typically chosen:

- The intercept is assigned a flat prior $p(\mu ) = \frac{1}{\sqrt{2 \cdot \pi } \sigma _M} e^{-\frac{\mu ^2}{2 \cdot \sigma _M^2}}$ with prior hyper-parameter $\sigma _M^2$ chosen to be very large to make the prior flat.
- The residual variance is assigned a scaled-inverse $\chi ^2$ density $p(\sigma ^2) = \chi ^{-2}(S_{\varepsilon }|\text {df}_{\varepsilon })$ with degrees of freedom parameter $\text {df}_{\varepsilon }$(> 0) and scale parameter $\text {S}_{\varepsilon }$(> 0).

The priors for the regression coefficients $\beta _{jk}$ can be chosen in different ways, for example, as flat priors similar to the intercept, which is considered an uninformative choice. Choosing informative priors not only provides a chance to introduce information on the coefficients known from previous runs of the study, but also allows performing penalized or regularized regression, such as Ridge regression or the LASSO through the choice of suitable priors.

Those coefficients utilizing flat priors are called “fixed” effects, as the estimation of the posterior is based only on information contained in the data itself, encoded by the likelihood. This is the reference model for regularised Bayesian models.

Choosing a Gaussian prior, according to [18], yields Ridge regression shrinkage estimation. Similar to [10] we call this approach the Bayesian ridge regression. Choosing double-exponential priors corresponds to the Bayesian LASSO model [10].

### Ensemble methods

Ensemble methods build multiple models using a given learning algorithm and then combine their predictions to produce an optimal estimate. The two most commonly used algorithms are *bagging* (or bragging) and *boosting*. Whereas *bagging* is a stagewise procedure that combines the predictions of multiple models (e.g., classification or regression trees) to yield an average prediction, *boosting* is a stagewise process in which each stage attempts to improve the predictions at the previous stage by up-weighting poorly predicted values. Below, we briefly discuss two popular ensemble methods, namely, random forests, an extension of bagging, and gradient boosting algorithms. Note that, although variable scaling (centering or standardizing) might accelerate convergence of the learning algorithms, the ensemble methods do not require it. Indeed, the collection of partition rules used with the ensemble methods should not change with scaling.

#### Random forests (RF)

The random forests algorithm is an ensemble algorithm that uses an ensemble of unpruned decision (classification or regression) trees, each grown using a bootstrap sample of the training data, and randomly selected (without replacement) subsets of the predictor variables (features) as candidates for splitting tree nodes. The randomness introduced by bootstrapping and selecting a random subset of the predictors reduces the variance of the random forest estimator, often at the cost of a slight increase in bias. The RF regression prediction for a new observation $y_i$, say $\widehat{y}_i^B$, is made by averaging the output of the ensemble of B trees $\{T(y_i,\Psi _b)\}_{b=1,...,B}$ as [56]

$$\begin{aligned} \widehat{y}_i^B = \frac{1}{B}\sum \limits _{b=1}^B T(y_i,\Psi _b) \end{aligned}$$

where $\Psi _b$ characterizes the *b*-th RF tree in terms of split variables, cut points at each node, and terminal node values. Recommendations on how to select the number of trees to grow, the number of covariates to be randomly chosen at each tree node and the minimum size of terminal nodes of trees, below which no split is attempted, are provided by [57, 58]. We refer to [56–58] for further details on the RF regression.

#### Stochastic gradient boosting (SGB)

Boosting enhances the predictive performance of base learners such as classification or regression trees, each of which performs only slightly better than random guessing, to become arbitrarily strong [56]. As with RF, boosting algorithms can also handle interactions, nonlinear relationships, automatically select variables and are robust to outliers, missing data and numerous correlated and irrelevant variables. In regression, boosting is an additive expansion of the form

$$\begin{aligned} {\textbf {y}}=f({\textbf {X}})=\sum \limits _{m=1}^M\beta _mh({\textbf {X}};\gamma _m) \end{aligned}$$

where $\beta _1,\dots ,\beta _M$ are the expansion coefficients and the basis functions $h({\textbf {X}};\gamma )$, base learners, are functions of the multivariate argument ${\textbf {X}}$, characterized by a set of parameters $\gamma =(\gamma _1,\dots ,\gamma _M)$. Typically these models are fit by minimizing a loss function *L* (e.g., the squared-error loss) averaged over the training data

$$\begin{aligned} \underset{\beta _m,\gamma _m}{\min }\sum \limits _{i=1}^nL\left( y_i,\sum \limits _{m=1}^M\beta _mh({\textbf {x}}_i;\gamma _m)\right) . \end{aligned}$$

We used regression trees as basis functions in which the parameters $\gamma _m$ are the splitting variables, split points at the internal nodes, and the predictions at the terminal nodes. Boosting regression trees involves generating a sequence of trees, each grown on the residuals of the previous tree. Prediction is accomplished by weighting the ensemble outputs of all the regression trees. We refer to [49, 56, 59] for further details on SGB (see, e.g., [59] for the interpretation of boosting in terms of regression for a continuous, normally distributed response variable).

### Instance-based methods

For the instance-based methods, scaling before applying the method is crucially important. Scaling the variables (features) prior to model fitting prevents possible numerical difficulties in the intermediate calculations and helps avoid domination of numeric variables with smaller by those with greater magnitude and range.

#### Support vector machines

Support vector machines (SVM) is a popular supervised learning technique for classification and regression of a quantitative response *y* on a set of predictors, in which case the method is called support vector regression or SVR [60]. In particular, SVR uses the model

14 $$\begin{aligned} y_i=f({\textbf {x}}_i)=\beta _0+h({\textbf {x}}_i)^T\varvec{\beta }, \end{aligned}$$

with ${\textbf {x}}_i=(x_{i1},\dots ,x_{ip})'$ and where the approximating function $f({\textbf {x}}_i)$ is a linear combination of basis functions $h({\textbf {x}}_i)^T$, which can be linear (or nonlinear) transformations of ${\textbf {x}}_i$. The goal of SVR is to find a function *f* such that $f({\textbf {x}}_i)$ deviates from $y_i$ by a value no greater than $\varepsilon$ for each training point ${\textbf {x}}_i$, and at the same time is as flat as possible. This so-called $\varepsilon$-insensitive SVR, or simply $\varepsilon$-SVR, thus fits a model (14) using only those residuals which are smaller in absolute value than $\varepsilon$ and a linear loss function for larger residuals. The choice of the loss function (e.g., linear, quadratic, Huber) usually considers the noise distribution pertaining to the data samples, level of sparsity and computational complexity.

If Eq. (14) is the usual linear regression model, i.e., $y_i=f({\textbf {x}}_i)=\beta _0+{\textbf {x}}_i^T\varvec{\beta }$, one considers the following minimization problem

15 $$\begin{aligned} \underset{\beta _0,\varvec{\beta }}{\min }\Big (\sum \limits _{i=1}^nV(y_i-f({\textbf {x}}_i))+\frac{\lambda }{2}\Vert \varvec{\beta }\Vert ^2\Big ) \end{aligned}$$

where $\lambda$ is the regularization parameter (cost) that controls the trade-off between flatness and error tolerance, $\Vert .\Vert$ refers to the norm under a Hilbert space (i.e., $\Vert \textbf{x} \Vert = \sqrt{\langle \textbf{x}{,} \textbf{x}\rangle }$ with $\textbf{x}$ a $p\ge 1$ dimensional vector) and

$$\begin{aligned} V_{\varepsilon }(r) = \left\{ \begin{array}{ll} 0, &{} \text {if}\ \vert r \vert < \varepsilon \\ \vert r \vert -\varepsilon , &{} \text {otherwise} \end{array}\right. \end{aligned}$$

is an $\varepsilon$-insensitive linear loss. Given the minimizers of (15) $\hat{\beta }_0$ and $\hat{\varvec{\beta }}$, the solution function has the form

$$\begin{aligned} \hat{\varvec{\beta }} = \sum \limits _{i=1}^n(\hat{\alpha }_i^*-\hat{\alpha }_i){\textbf {x}}_i \quad \text { and }\quad \hat{f}({\textbf{x}}) = \sum \limits _{i=1}^n(\hat{\alpha }_i^*-\hat{\alpha }_i)\langle {\textbf{x}}{,} {\textbf {x}}_i\rangle +\hat{\beta _0} \end{aligned}$$

where $\hat{\alpha }^*_i, \ \hat{\alpha }_i$ are positive weights given to each observation (i.e., to the column vector ${\textbf{x}}_i$) estimated from the data. Typically only a subset of $(\hat{\alpha }_i^*-\hat{\alpha }_i)$ are non-zero with the observations associated to these so called *support vectors*, and thus the name of the method, SVM. More details on SVM can be found in [56].

### Deep learning methods

Deep learning (DL) algorithms are implemented through neural networks, which encompass an assortment of architectures (e.g., convolutional, recurrent and densely connected neural networks) and depend on many parameters and hyperparameters whose careful optimization is crucial to enhancing predictive accuracy and minimizing overfitting (see [8, 61–65] for further insights into DL architectures and other particulars and the supplementary materials https://github.com/miguelperezenciso/DLpipeline of [8] for a list of the main DL hyperparameters, their role and related optimization issues). It can be very challenging to achieve great improvements in predictive accuracy in genomic prediction studies with DL because hyperparameter optimization can be extremely demanding and also because DL requires very large training datasets which might not always be available [1–4].

After selecting a DL architecture there is usually a large set of parameters to be set in order to minimize some fitting criterion such as least squares or some measure of entropy from some training data (network training). Therefore, an optimization method must also be selected. The three top ranked optimizers for neural networks are mini-batch gradient descent, gradient descent with momentum and adaptive moment estimation (ADAM; [66]). Among the three, the mini-batch gradient descent and Adam are usually preferred, because they perform well most of the time. In terms of convergence speed, ADAM is often clearly the winner and thus a natural choice [67].

**Fig. 1:**
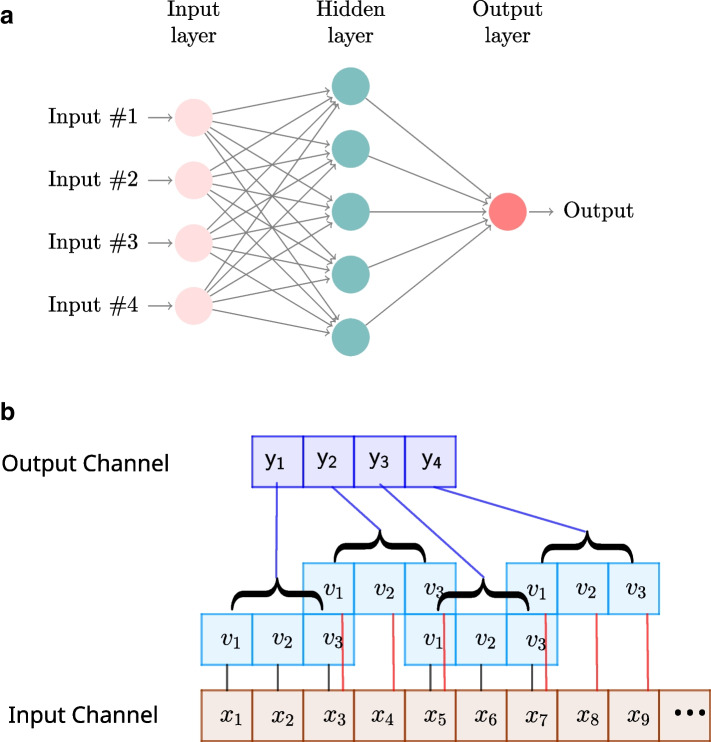
Graphical representation of **a** a feed-forward neural network (FFNN) with one hidden layer; and **b** a convolution of a filter $(v_1,v_2,v_3)$, with stride=2, on the Input Channel $(x_1,x_2,\dots )$. The result is in the Output Channel $(y_1,y_2,\dots )$

Next, we offer a few more details on the feed-forward and convolutional neural networks, which, besides being some of the most popular DL architectures, are well suited for regression problems. These models can be represented graphically as a set of inputs linked to the outputs through one or more hidden layer. Figure 1a represents such a model (either FFNN or CNN) with a single hidden layer.

Further details on neural networks in general and FFNN and CNN in particular can be found in [1–4, 8, 56]. Note that, to avoid potential numerical difficulties, it is recommended that both the target (response variable; here assumed to be continuous and normally distributed), and the features (covariates) are standardized prior to training the network [8].

#### Feed-forward neural network (FFNN)

A feed-forward neural network (FFNN), also known in the literature as a multi-layer perceptron (MLP), is a neural network that does not assume a specific structure in the input features (i.e., in the covariates). This neural network consists of an input layer, an output layer and multiple hidden layers between the input and output layers.

The model for a FFNN with one hidden layer expressed as a multiple linear regression model (1) is given by

$$\begin{aligned} y_i=\alpha +\sum \limits _h w_{h}\phi \Big (\alpha _h+\sum \limits _j w_{jh}x_{ij}\Big ) \end{aligned}$$

where the $y_i$ (output) and $x_{ij}$ (input) are defined as in model (1), $\alpha$ is the output bias, *h* runs over the units of the hidden layer, $\alpha _h$ refers to the bias of the *h*-th unit of the hidden layer, $w_{jh}$ refer to the weights between the inputs and the hidden layer, $w_h$ refer to the weights between the hidden layer and the output, $\phi$ is the *activation* function of the hidden layer. The model parameters $\alpha$, $\alpha _h$, $w_h$ and $w_{jh}$ are unknown network parameters that need to be estimated in the network training process.

#### Convolutional neural network (CNN)

A convolution neural network (CNN) is a neural network that contains one or more convolution layers, which are defined by a set of filters. Although a CNN generally refers to a 2-dimensional neural network, which is used for image analysis, in this study we consider a 1-dimensional (1D) CNN. Here, the input to the 1D convolution layer is a vector ${\textbf{x}}=(x_1,\dots ,x_p)$ equal to one row of the $n\times p$ marker matrix $\textbf{X}$. The 1D convolution filter is defined by a vector ${\textbf{v}}=(v_1,\dots ,v_d)$ where $d<p$. The convolution of a filter ${\textbf{v}}$ with ${\textbf{x}}$, which is called a *channel*, is a vector ${\textbf{y}}=(y_1,y_2,\dots )$ satisfying

$$\begin{aligned} y_i=x_{1+s(i-1)}v_1+x_{2+s(i-1)}v_2+\dots ++x_{d+s(i-1)}v_d\,\, \end{aligned}$$

where *s*, i.e., the stride length, is the shift displacement of the filter across the input data. An activation function is applied after each convolution to produce an output. Figure 1b depicts a 1D convolution of a filter $(v_1,v_2,v_3)$ on the input vector $(x_1,x_2,\dots ,x_9,\dots )$, considering a stride of length $s=2$, which results in the output channel $(y_1,y_2,\dots )$. Filter values $v_1,\dots , v_d$ are model parameters that are estimated in the neural network training process.

### Performance assessment

For the simulated dataset, we assessed predictive performance using predictive accuracy (PA), the Pearson correlation between the predicted (PGBVs) and the simulated true (TGBVs) breeding values. For all the three KWS empirical data sets, predictive performance was expressed as predictive ability (PA), the Pearson correlation between the PGBVs and the observed (adjusted means estimated from phenotypic analysis) genomic breeding values (OGBVs), also calculated using cross validation. The simulated true breeding values are specified in the simulation model and therefore are known exactly. In contrast, for empirical data, the true breeding values are unknown and are approximated by the observed breeding values estimated as adjusted means during phenotypic analysis. The higher the PA, the better is the relative predictive performance of a method. We additionally assessed the predictive performance of the methods using the out-of-sample mean squared prediction error (MSPE) and the mean absolute prediction error (MAPE). Specifically,

$$\begin{aligned} \text {PA} = \frac{\sum \limits _i(y_i-\bar{y})(\hat{y}_i-\bar{\hat{y}})}{\sqrt{\sum \limits _i(y_i-\bar{y})^2\sum \limits _i(\hat{y}_i-\bar{\hat{y}})^2}},\quad \text {MSPE}=\frac{1}{n}\sum \limits _i(y_i-\hat{y}_i)^2,\quad \text {MAPE}=\frac{1}{n}\sum \limits _i\vert y_i-\hat{y}_i \vert , \end{aligned}$$

where the $y_i$ and $\bar{y}$ are, respectively, the TGBVs and mean TGBVs for the single simulated dataset, but the OGBVs and mean OGBVs for the empirical datasets, and the $\hat{y}_i$ and $\bar{\hat{y}}_i$ are, respectively, the PGBVs and mean PGBVs. 10-fold CV is used to assess the PA for each method for the simulated datasets in contrast to the 5-fold CV used with the three empirical maize datasets. Although we report both the prediction errors and the PA, breeders are primarily interested in the final ordering of the genotypes, which the PA captures better than the prediction errors.

For the cross validation, we aimed to have at least 150 individuals per fold. Accordingly, each phenotypic dataset was randomly split into *k* approximately equal parts. The breeding values for each of the *k* folds were predicted by training the model on the $k-1$ remaining folds and a CV error (CVE) computed for each of the *k* folds. The method with the smallest CVE was selected to predict the breeding values for the unphenotyped genotypes for the simulated dataset, and the phenotyped genotypes in the validation sets for each of the three empirical maize datasets.

All the methods are implemented in the R software and are available in various R packages [10, 32, 40, 43, 48, 54, 58, 68–73]. Table S1 (Additional file 5, Section 3) lists the R packages we used to analyse the synthetic and real datasets. For the deep learning methods, and because of fine tuning requirements, we used the Python software and packages Numpy, Pandas and Tensorflow [74, 75]. All R and Python codes referring to the simulated data are provided in Additional files 2 & 3.

Noteworthy details of model fitting are available in the supplementary materials (Additional file 5, Section 2).

## Results

Although we did not fully quantify the computational costs of the different methods, the computational burden increased strikingly from the simple regularized through the adaptive to the grouped methods. A similar trend was also apparent from the ensemble, through the instance-based to the deep learning methods. Computational time may be reduced greatly by parallelizing the estimation or optimization algorithms, but this strategy may not always be available and can be challenging to implement for some methods.

### Simulated (animal) data

**Fig. 2:**
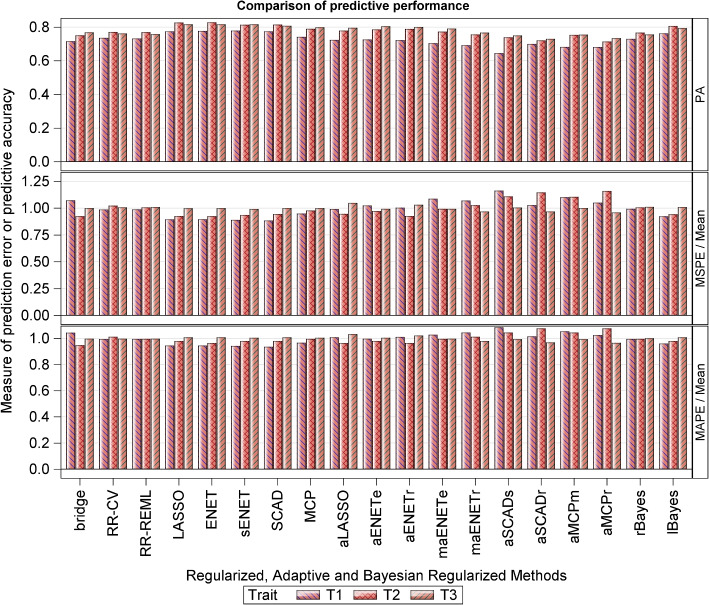
Prediction accuracy (PA) of the regularized, adaptive regularized and Bayesian regularized methods, computed as the Pearson correlation coefficient between the true breeding values (TBVs) and the predicted breeding values (PBVs), for the simulated dataset, where $T_1-T_3$ refer to three quantitative milk traits. The choice of $\lambda$, where applicable, was based on the 10-fold CV. The mean squared and absolute prediction errors are also provided. See Table S2 for details

**Fig. 3:**
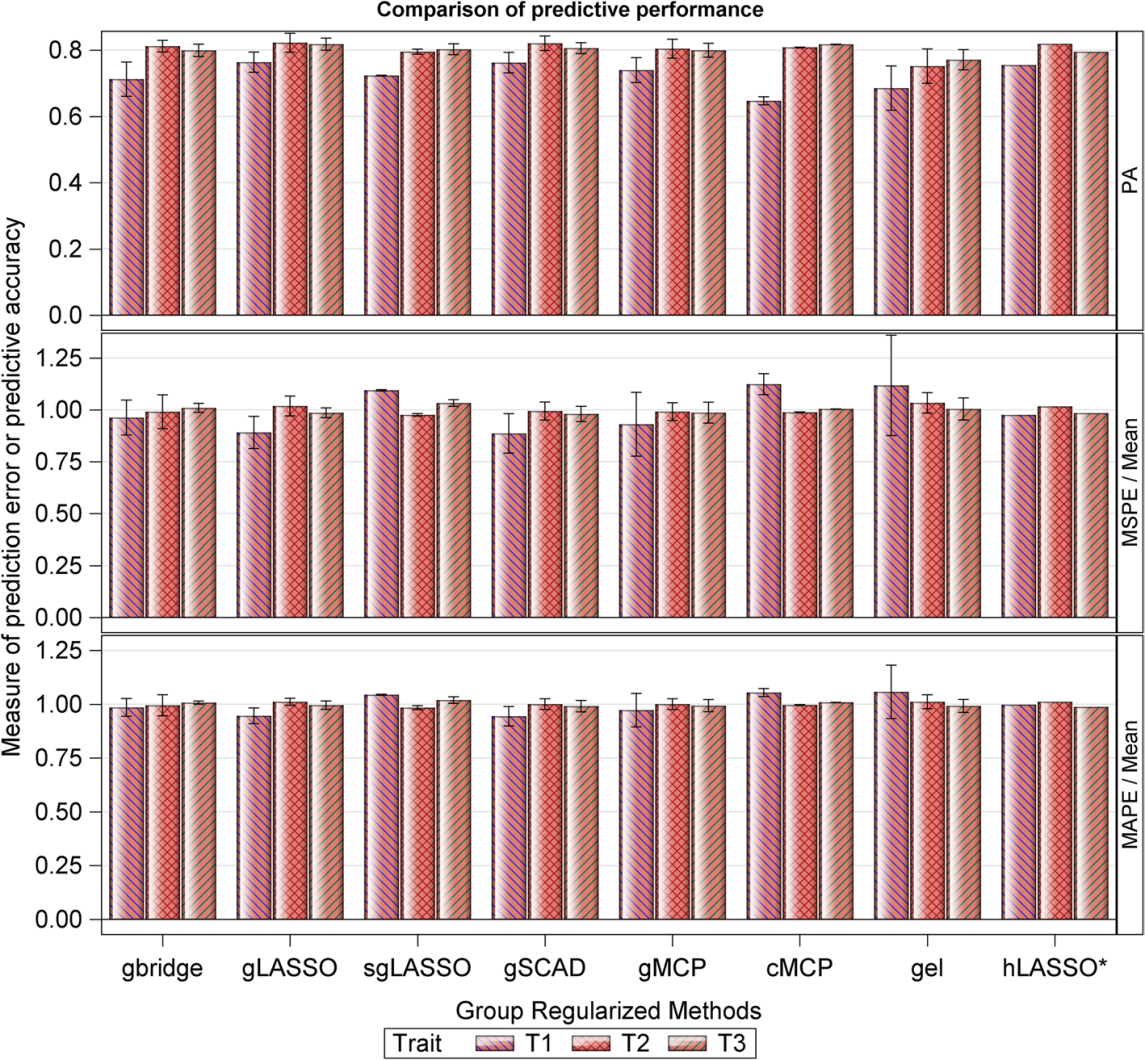
Prediction accuracy (PA) of the group regularized methods (mean and range values of PA across the different groupings), computed as the Pearson correlation coefficient between the true breeding values (TBVs) and the predicted breeding values (PBVs), for the simulated dataset, where $T_1-T_3$ refer to three quantitative milk traits. Choice of $\lambda$ was based on the 10-fold CV. Display refers to the mean, max and min values of PA across all the 10 grouping schemes. The mean squared and absolute prediction errors are also provided. See Table S3 for details

**Fig. 4:**
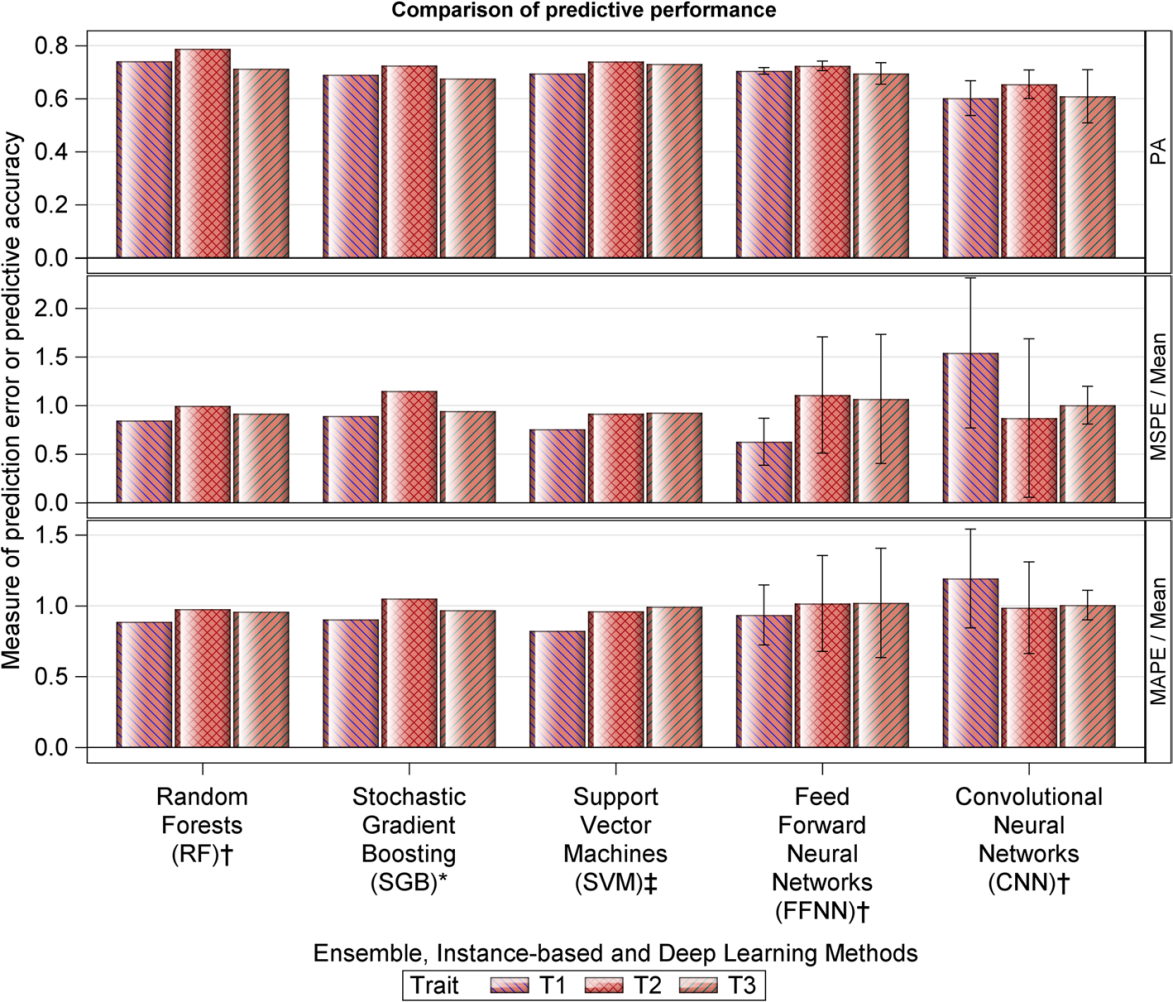
Prediction accuracy (PA) of the ensemble, instance-based and deep learning methods, computed as the Pearson correlation coefficient between the true breeding values (TBVs) and the predicted breeding values (PBVs), for the simulated dataset, where $T_1-T_3$ refer to three quantitative milk traits. See Tables S4-S5 for details

The relative performances of the various methods on the simulated data varied with the target trait and with whether performance was assessed in terms of predictive accuracy or prediction error. Performance also varied in terms of computational cost with some methods requiring considerably more time than others. Results of genomic prediction accuracy for the simulated data are displayed in Figs. 2, 3 and 4 and Tables S2-S5 (Additional file 5, Section 3). Tables S6 & S7 (Additional file 5, Section 3) report the calibration details for the fitted feed-forward and convolutional neural networks.

Table 6 displays the range of the observed predictive accuracies across all the classes of the regularized methods for traits $T_1-T_3$. Neither the adaptive, group, nor Bayesian regularized methods seem to improve upon the results of their regularized counterparts, although group regularized methods do provide some slight improvement upon the results of the adaptive regularized methods. Even though all the regularized regression methods had comparable overall performance, the best compromise between high PA ($\ge 0.77$ for $T_1$, 0.82 for $T_2$ and 0.81 for $T_3$) and small prediction errors was achieved by the LASSO, ENET, sENET and SCAD (Fig. 2 and Table S2; first half). Within the class of adaptive regularized methods, the best compromise was achieved by aLASSO and aENET (Fig. 2 and Table S2; second half; PA$\ge 0.72$ for $T_1$, 0.78 for $T_2$ and 0.80 for for $T_3$). For the group regularized methods, a good compromise was achieved by the gLASSO and gSCAD (Fig. 2 and Table S2; mean PA values $\ge 0.76$ for $T_1$, 0.82 for $T_2$ and 0.81 for $T_3$). Whereas the worst performing group regularized methods in terms of the estimated PAs were the cMCP and gel for $T_1$ (PA$<0.7$), sgLASSO and gel for $T_2$ (PA$<0.8$) and hLASSO and gel for $T_3$ (PA$<0.8$), the worst performing methods in terms of prediction errors were the gel ($T_1$ & $T_2$ only) and sgLASSO ($T_3$ only). Of all the group regularized methods, the most time consuming were the sgLASSO and hLASSO, with sgLASSO requiring several more months to compute results for trait $T_1$ than for traits $T_2$ or $T_3$. In the comparisons between the two Bayesian regularized methods, Lasso Bayes consistently outperformed the Ridge Bayes method across all the three traits, demonstrating superior predictive accuracy and generally smaller prediction errors.

**Table 6:** Range of the estimated predictive accuracies across the classes of regularized methods for traits $T_{1}-T_{3}$

	$T_1$	$T_2$	$T_3$
Regularized	$0.716-0.779$	$0.770-0.829$	$0.758-0.817$
Adaptive Regularized	$0.645-0.726$	$0.714-0.789$	$0.730-0.805$
Group Regularized^†^	$0.653-0.766$	$0.758-0.820$	$0.765-0.814$
Bayesian Regularized	$0.730-0.763$	$0.767-0.807$	$0.756-0.794$

^†^ Values refer to the range of the observed mean PAs

The ensemble, instance-based and deep learning methods did not improve upon the results of the regularized, the group or the Bayesian regularized methods (Fig. 4 and Tables S4 & S5). Among the ensemble and instance-based groups of methods, RF provided the best compromise between high PA and small prediction errors. For the deep learning methods, the FFNN provided consistently higher PA values than CNN across all the three traits from the simulated data.

Predictive performance varied not only among the methods but also with the target quantitative traits. Specifically, trait $T_3$ had the highest predictive accuracies for the adaptive methods, whereas trait $T_2$ was generally top ranked across all the remaining methods.

### Real (plant) data

The ridge regression methods plus the overall best performing methods (high PA values and low prediction errors) for each class of methods based on the analysis of the simulated dataset, were applied to each of the three KWS empirical maize datasets. The specific methods fitted to the KWS maize datasets comprised RR-CV, RR-REML, sENET, aENET (enet penalty), gLASSO, RF, FFNN and lBayes.

**Fig. 5:**
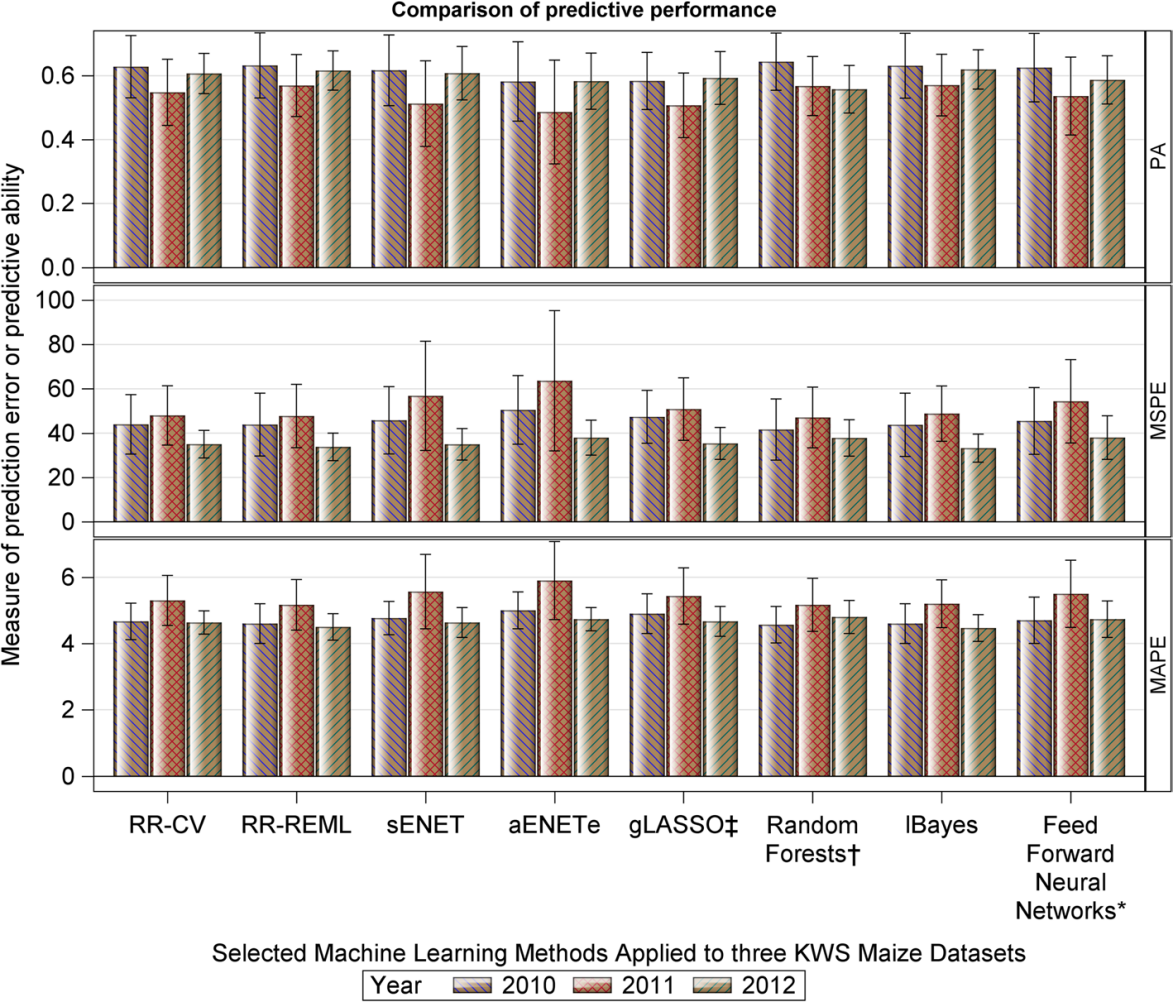
Predictive ability (PA; mean and range values computed across the 5-fold validation datasets and 10 replicates) of the regularized and adaptive regularized methods, computed as the Pearson correlation coefficient between the observed breeding values (OBVs) and the predicted breeding values (PBVs), for the KWS datasets. The choice of $\lambda$, where applicable, was based on 4-fold CV. See Table S8 for details

Results are displayed in Fig. 5 and Table S8 (Additional file 5, Section 3). Across the three real maize datasets, the highest predicitive abilities were obtained for the 2010 dataset. The estimated predictive abilities (PA) are under 0.7 for the 2010 dataset but under 0.6 for the 2011 dataset and generally under 0.6 for the 2012 dataset (RR-REML and lBayes excluded with estimated PAs of 0.616 and 0.624, respectively), regardless of the method used. The lBayes and RR-REML (2011 & 2012 datasets) and RF, RR-REML and lBayes (2010 dataset) are evidently the best performing methods (higher PA values and lower prediction errors). On the other hand, aENET^e^ (2010 & 2011 datasets) and RF (2012 dataset) are the worst performing methods (lower PA and higher prediction errors). Interestingly, the RF performed both the best (2010 dataset) and the worst (2012 dataset), clearly emphasizing that the methods are strongly data dependent.

## Discussion

We have investigated the predictive performance of several state-of-the art machine learning methods in genomic prediction via the use of one simulated and three real datasets. All the methods showed reasonably high predictive performance for most practical selection decisions. But the relative predictive performance of the methods was both data and target trait dependent, complicating and precluding omnibus comparative evaluations of the genomic prediction methods, thus ruling out selection of one procedure for routine use in genomic prediction. These results broaden the findings of earlier studies (e.g. [9]) to encompass a wider range of groups of methods. If reproducibility of results, low computational cost and time are important considerations, then using the regularized regression methods comes highly recommended because they consistently produced, with relatively lower computational cost and computing time, reasonably accurate and competitive predictions relative to the other groups of methods for the simulated and the three real datasets. Even among the regularized regression methods, increasing model complexity from simple through the adaptive to grouped or even the Bayesian regularized methods, generally only increased computing time without clearly improving predictive performance.

The ensemble, instance-based and deep-learning ML methods need the tuning of numerous hyperparameters thus requiring considerable computing time to adequately explore the entire hyperparameter space. This will not always be possible in most applications because of limiting time and computational resources leading to potentially less than optimal results and may well partly explain why these methods did not clearly outperform the classical ML methods. Indeed, the computational costs of the ensemble, instance-based and deep learning methods can quickly become prohibitive, if all the parameters are tuned by searching over the often large grid of values. This will typically require not only proficiency in programming and algorithm parallelization and optimization, but excellent computing resources. These constraints, plus the growing size of phenotypic and genomic data, make it difficult to identify methods for routine use in genomic prediction and call for greater focus on and investment in enhancing the computational efficiencies of algorithms and computing resources.

We have considered only well tested and established off-the-shelf machine learning methods and one simulated and three real datasets. We are extending this work to cover the following four objectives. (1) Comparing the performance of methods that use advanced techniques for feature selection or dimensionality reduction on multiple synthetic datasets simulated using different configurations or scenarios. (2) Exploring how the methods generalize based on different training/test splits across simulations/real-world datasets, individuals/samples, or chromosomes. (3) Evaluating the sensitivity of the different methods to hyperparameter selection. (4) Assessing the training and testing complexity for the different methods.

## Conclusions

Machine learning methods are well suited for efficiently handling high dimensional data. Particularly, supervised machine learning methods have been successfully used in genomic prediction or genome-enabled selection. However, their comparative predictive accuracy is still poorly understood, yet this is a critical issue in plant and animal breeding studies given that increasing methodological complexity can substantially increase computational complexity or cost. Here, we showed that predictive performance is both data and target trait dependent thus ruling out selection of one method for routine use in genomic prediction. We also showed that for this reason, relatively low computational complexity and competitive predictive performance, the classical linear mixed model approach and regularized regression methods remain strong contenders for genomic prediction.

### Supplementary Information

**Additional file 1.** Simulated (animal breeding) dataset. Includes four txt files: one for the grouping schemes, one for the QTLMAS prediction data, one for the QTLMAS training data, and one for the validation trait values.

**Additional file 2.** R codes used to fit the ML algorithms to the simulated (animal breeding) dataset. Includes six R files: one for the simple regularized methods, one for the adaptive regularized methods, one for the group regularized methods, one for the Bayesian regularized methods, one for the ensemble methods, and one for the instance-based methods.

**Additional file 3.** Python codes used to fit the deep learning (FFNN & CNN) algorithms to the simulated (animal breeding) dataset. Includes six py and three pnz files: three of the py files refer to the FFNN fits and the other three to the CNN fits; each of the three pnz files include six npy files referring to the training of the FFNNs for traits 1, 2 & 3, respectively.

**Additional file 4.** Includes SAS code for (i) the phenotypic data analysis (S1 Text.doc); (ii) SNP grouping schemes (S2 Text.doc); and (iii) the 5-fold data split (S3 Text.doc & S4 Text.doc) for the KWS $2010-2012$ data sets.

**Additional file 5.** Includes the RR-BLUP model used to estimate variance components for the KWS real maize data (Section 1), the Noteworthy details of model fitting (Section 2) plus the additional Tables of results (Section 3). **Table S1.** List of R and Python packages used in this paper. **Table S2.** Prediction accuracy (PA) of the regularized, adaptive regularized and Bayesian regularized methods, computed as the Pearson correlation coefficient between the true breeding values (TBVs) and the predicted breeding values (PBVs), for the simulated dataset, where $T_1-T_3$ refer to three quantitative milk traits. The choice of $\lambda$, where applicable, was based on the 10-fold CV. The mean squared and absolute prediction errors are also provided. **Table S3.** Prediction accuracy (PA) of the group regularized methods (mean and range values of PA across the different groupings), computed as the Pearson correlation coefficient between the true breeding values (TBVs) and the predicted breeding values (PBVs), for the simulated dataset, where $T_1-T_3$ refer to three quantitative milk traits. Choice of $\lambda$ was based on the 10-fold CV. Display refers to the mean, max and min values of PA across all the 10 grouping schemes. The mean squared and absolute prediction errors are also provided. **Table S4.** Prediction accuracy (PA) of the ensemble and instance-based methods, computed as the Pearson correlation coefficient between the true breeding values (TBVs) and the predicted breeding values (PBVs), for the simulated dataset, where $T_1-T_3$ refer to three quantitative milk traits. **Table S5.** Prediction accuracy (PA) of the deep learning methods, computed as the Pearson correlation coefficient between the true breeding values (TBVs) and the predicted breeding values (PBVs), for the simulated dataset, where $T_1-T_3$ refer to three quantitative milk traits. **Table S6.** Best FFNN model calibration parameters selected for each of the three quantitative milk traits $T_1-T_3$. **Table S7.** Best CNN model calibration parameters (Number of epochs/Learning rate) selected for each of the three quantitative milk traits $T_1-T_3$. **Table S8.** Predictive ability (PA; mean and range values computed across the 5-fold validation datasets and 10 replicates) of the regularized, adaptive regularized, group regularized, Bayesian regularized, ensemble, instance-based and deep learning methods, computed as the Pearson correlation coefficient between the observed breeding values (OBVs) and the predicted breeding values (PBVs), for the KWS datasets. The choice of $\lambda$, where applicable, was based on 4-fold CV.

## Data Availability

The simulated animal data from the QTLMAS workshop 2012 is provided in the supplementary materials together with the annotated R and Python codes used to analyse these data. The KWS data is proprietary data and cannot be shared publicly for confidentiality reasons. These can only be shared upon reasonable request and with KWS' express consent. This notwithstanding, we provide a synthetic dataset that mimics the KWS data, which can be used with our codes to illustrate the implementation of the ML methods.

## Appendix

### Observation for $\widehat{\varvec{\beta }}_{BLUP}$ derivation:

$$\begin{aligned} ({\textbf {X}}'{} {\textbf {X}}+{\textbf {H}})^{-1}{} {\textbf {X}}'({\textbf {K}}+{\textbf {H}}){\textbf {K}}^{-1}{} {\textbf {X}}&= ({\textbf {X}}'{} {\textbf {X}}+{\textbf {H}})^{-1}({\textbf {X}}'{} {\textbf {X}}{} {\textbf {X}}'+{\textbf {X}}'{} {\textbf {H}}){\textbf {K}}^{-1}{} {\textbf {X}}\\&=({\textbf {X}}'{} {\textbf {X}}+{\textbf {H}})^{-1}({\textbf {X}}'{} {\textbf {X}}+{\textbf {H}}){\textbf {X}}'{} {\textbf {K}}^{-1}{} {\textbf {X}}={\textbf {X}}'{} {\textbf {K}}^{-1}{} {\textbf {X}} \end{aligned}$$

### Observation for $\widehat{\textbf{g}}_{BLUP}$ derivation:

$$\begin{aligned} {\textbf {X}}({\textbf {X}}'{} {\textbf {X}}+{\textbf {H}})^{-1}{} {\textbf {X}}'({\textbf {K}}+{\textbf {H}}){\textbf {K}}^{-1}&= {\textbf {X}}({\textbf {X}}'{} {\textbf {X}}+{\textbf {H}})^{-1}({\textbf {X}}'{} {\textbf {X}}{} {\textbf {X}}'+{\textbf {X}}'{} {\textbf {H}}){\textbf {K}}^{-1}\\&={\textbf {X}}({\textbf {X}}'{} {\textbf {X}}+{\textbf {H}})^{-1}({\textbf {X}}'{} {\textbf {X}}+{\textbf {H}}){\textbf {X}}'{} {\textbf {K}}^{-1}={\textbf {I}} \end{aligned}$$

## Abbreviations

ADAM: Adaptive moment estimation
BLUP: Best linear unbiased prediction
CV: Cross-validation
DL: Deep learning
ENET: Elastic net
FFNN: Feed-forward neural network
GP: Genomic prediction
GS: Genomic selection
LASSO: Least absolute shrinkage and selection operator
MAPE: Mean absolute prediction error
MCP: Minimax concave penalty
ML: Machine learning
MLP: Multi-layer perceptron
MSPE: Mean squared prediction error
OLS: Ordinary least squares
PA: Predictive accuracy/ability
PCA: Principal component analysis
PGBV: Predicted genomic breeding value
QTL: Quantitative trait loci
REML: Restricted maximum likelihood
RF: Random forests
RR: Ridge regression
RSS: Residual sum of squares
SCAD: Smoothly clipped absolute deviation
SGB: Stochastic gradient boosting
SNP: Single nucleotide polymorphism
TGBV: True genomic breeding value
SVM: Support vector machine
SVR: Support vector regression

## References

[1] Montesinos-López A, Montesinos-López OA, Gianola D, Crossa J, Hernández-Suárez CM. Multi-environment genomic prediction of plant traits using deep learners with dense architecture. G3 Genes Genomes Genet. 2018;8(12):3813–3828. 10.1534/g3.118.200740 PMC6288841 30291107

[2] Montesinos-López OA, Montesinos-López A, Crossa J, Gianola D, Hernández-Suárez CM, Martín-Vallejo J. Multi-trait, multi-environment deep learning modeling for genomic-enabled prediction of plant traits. G3 Genes Genomes Genet. 2018;8(12):3829–3840. 10.1534/g3.118.200728 PMC6288830 30291108

[3] Montesinos-López OA, Martín-Vallejo J, Crossa J, Gianola D, Hernández-Suárez CM, Montesinos-López A, Philomin J, Singh R. A benchmarking between deep learning, support vector machine and Bayesian threshold best linear unbiased prediction for predicting ordinal traits in plant breeding. G3 Genes Genomes Genet. 2019;9(2):601–618. 10.1534/g3.118.200998 PMC6385991 30593512

[4] Montesinos-López OA, Martín-Vallejo J, Crossa J, Gianola D, Hernández-Suárez CM, Montesinos-López A, Juliana P, Singh R. New deep learning genomic-based prediction model for multiple traits with binary, ordinal, and continuous phenotypes. G3 Genes Genomes Genet. 2019;9(5):1545–1556. 10.1534/g3.119.300585 PMC6505163 30858235

[5] Ogutu JO, Piepho H-P, Schultz-Streeck T. A comparison of random forests, boosting and support vector machines for genomic selection. BMC Proc. 2011;5(3):1-5. 10.1186/1753-6561-5-S3-S11 PMC3103196 21624167

[6] Ogutu JO, Schulz-Streeck T, Piepho H-P. Genomic selection using regularized linear regression models: ridge regression, lasso, elastic net and their extensions. BMC Proc. 2012;6(2):1-6. 10.1186/1753-6561-6-S2-S10 PMC3363152 22640436

[7] Heslot N, Yang HP, Sorrells ME, Jannink JL (2012). Genomic selection in plant breeding: a comparison of models. Crop Sci..

[8] Pérez-Enciso M, Zingaretti LM (2019). A Guide on Deep Learning for Complex Trait Genomic Prediction. Genes..

[9] Ogutu JO, Piepho H-P. Regularized group regression methods for genomic prediction: Bridge, MCP, SCAD, group bridge, group lasso, sparse group lasso, group MCP and group SCAD. BMC Proc. 2014;8(5):1-9. 10.1186/1753-6561-8-S5-S7 PMC4195413 25519521

[10] Pérez P, de los Campos G. Genome-wide regression and prediction with the BGLR statistical package. Genetics. 2014;198:483–495. 10.1534/genetics.114.164442 PMC4196607 25009151

[11] Usai MG, Gaspa G, Macciotta NP, Carta A, Casu S. XVIth QTLMAS: simulated dataset and comparative analysis of submitted results for QTL mapping and genomic evaluation. BMC Proc. 2014;8(5):1–9. 10.1186/1753-6561-8-S5-S1 PMC4195410 25519515

[12] Estaghvirou SBO, Ogutu JO, Schulz-Streeck T, Knaak C, Ouzunova M, Gordillo A, Piepho HP (2013). Evaluation of approaches for estimating the accuracy of genomic prediction in plant breeding. BMC Genomics..

[13] Estaghvirou SBO, Ogutu JO, Piepho HP (2015). How genetic variance and number of genotypes and markers influence estimates of genomic prediction accuracy in plant breeding. Crop Sci..

[14] Xie L. Randomly split SAS data set exactly according to a given probability Vector. 2009. https://silo.tips/download/randomly-split-sas-data-set-exactly-according-to-a-given-probability-vector. Accessed 15 Mar 2021.

[15] Frank IE, Friedman JH (1993). A statistical view of some chemometrics regression tools (with discussion). Technometrics..

[16] Fan J, Li R (2001). Variable selection via nonconcave penalized likelihood and its oracle properties. J Am Stat Assoc..

[17] Fan J, Peng H (2004). Nonconcave penalized likelihood with a diverging number of parameters. Ann Stat..

[18] Hoerl AE, Kennard RW (1970). Ridge regression: biased estimation for non-orthogonal problems. Technometrics..

[19] Tibshirani R (1996). Regression shrinkage and selection via the lasso. J R Stat Soc B..

[20] Zou H, Hastie T (2005). Regularization and variable selection via the elastic net. J R Stat Assoc B..

[21] Fu WJ (1998). Penalized regressions: The bridge versus the lasso. J Comput Graph Stat..

[22] Huang J, Horowitz JL, Ma S (2008). Asymptotic properties of bridge estimators in sparse high-dimensional regression models. Ann Stat..

[23] Knight K, Fu W (2000). Asymptotics for Lasso-type estimators. Ann Stat..

[24] Zhang C-H, Huang J (2008). The sparsity and bias of the lasso selection in high-dimensional linear regression. Ann Stat..

[25] Zhang C-H (2010). Nearly unbiased variable selection under minimax concave penalty. Ann Stat..

[26] Meuwissen TH, Hayes BJ, Goddard M (2001). Prediction of total genetic value using genome-wide dense marker maps. Genetics..

[27] Searle SR, Casella G, McCulloch CE (1992). Variance components.

[28] Piepho H-P, Ogutu JO, Schulz-Streeck T, Estaghvirou B, Gordillo A, Technow F (2012). Efficient computation of ridge-regression best linear unbiased prediction in genomic selection in plant breeding. Crop Sci..

[29] Ruppert D, Wand MP, Carroll RJ. Semiparametric regression. Cambridge: Cambridge University Press; 2003.

[30] Hayes BJ, Visscher PM, Goddard ME (2009). Increased accuracy of artificial selection by using the realized relationship matrix. Genet Res..

[31] Piepho H-P (2009). Ridge regression and extensions for genomewide selection in maize. Crop Sci..

[32] Mazumder R, Friedman JH, Hastie T (2011). Sparsenet: Coordinate descent with nonconvex penalties. J Am Stat Assoc..

[33] Kim Y, Choi H, Oh HS (2008). Smoothly clipped absolute deviation on high dimensions. J Am Stat Assoc..

[34] Zhang C-H. Penalized linear unbiased selection. Department of Statistics and Bioinformatics, Rutgers University, Technical Report #2007-003. 2007.

[35] Breheny P, Huang J (2011). Coordinate descent algorithms for nonconvex penalized regression, with applications to biological feature selection. Ann Appl Stat..

[36] Chen Z, Zhu Y, Zhu C (2016). Adaptive bridge estimation for high-dimensional regression models. J Inequalities Appl..

[37] Zou H (2006). The adaptive lasso and its oracle properties. J Am Stat Assoc..

[38] Grandvalet Y. Least absolute shrinkage is equivalent to quadratic penalization. International Conference on Artificial Neural Networks. London: Springer; 1998. p. 201–206.

[39] Zou H, Zhang HH (2009). On the adaptive elastic-net with a diverging number of parameters. Ann Stat..

[40] Xiao N, Xu QS (2015). Multi-step adaptive elastic-net: reducing false positives in high-dimensional variable selection. J Stat Comput Simul..

[41] Huang J, Breheny P, Ma S. A Selective Review of Group Selection in High-Dimensional Models. Stat Sci. 2012;27(4). 10.1214/12-STS392. 10.1214/12-STS392 PMC3810358 24174707

[42] Bach F (2008). Consistency of the group lasso and multiple kernel learning. J Mach Learn..

[43] Breheny P, Huang J (2009). Penalized methods for bi-level variable selection. Stat Interface..

[44] Park C, Yoon YJ (2011). Bridge regression: adaptivity and group selection. J Stat Plan Infer..

[45] Yuan M, Lin Y (2006). Model selection and estimation in regression with grouped variables. J R Stat Soc B..

[46] Breheny P, Huang J (2015). Group descent algorithms for nonconvex penalized linear and logistic regression models with grouped predictors. Stat Comput..

[47] Huang J, Ma S, Xie H, Zhang C-H (2009). A group bridge approach for variable selection. Biometrika..

[48] Simon N, Friedman J, Hastie T, Tibshirani R. A sparse-group lasso. J Comput Graph Stat. 2013;22:231–45. 10.1080/10618600.2012.681250.

[49] Friedman J, Hastie T, Tibshirani R. A note on the group lasso and sparse group lasso. 2010. arXiv preprint arXiv:1001.0736.

[50] Huang J, Zhang T (2010). The benefit of group sparsity. Ann Stat..

[51] Poignard B (2020). Asymptotic theory of the adaptive Sparse Group Lasso. Ann Inst Stat Math..

[52] Percival D (2011). Theoretical properties of the overlapping groups lasso. Electron J Stat..

[53] Zhou N, Zhu J (2010). Group variable selection via a hierarchical lasso and its oracle property. Stat Interface..

[54] Lim M, Hastie T (2015). Learning interactions via hierarchical group-lasso regularization. J Comput Graph Stat..

[55] Bien J, Taylor J, Tibshirani R (2013). A lasso for hierarchical interactions. Ann Stat..

[56] Hastie TJ, Tibshirani R, Friedman J (2009). The elements of statistical learning.

[57] Liaw A, Wiener M (2002). Classification and regression by randomForest. R News..

[58] Breiman L (2001). Random forests. Mach Learn..

[59] Schonlau M (2005). Boosted regression (boosting): An introductory tutorial and a Stata plugin. Stata J..

[60] Vapnik V (1995). The Nature of Statistical Learning Theory.

[61] Min S, Lee B, Yoon S. Deep learning in bioinformatics. Brief Bioinforma. 2017;18(5):851–69. 10.1093/bib/bbw068. 10.1093/bib/bbw068 27473064

[62] Yue T, Wang H. Deep learning for genomics: A concise overview. 2018. arXiv preprint arXiv:1802.00810.

[63] Bengio Y. Practical recommendations for gradient-based training of deep architectures. In: Neural Networks: Tricks of the trade. Berlin, Heidelberg: Springer; 2012. p. 437–478.

[64] Eraslan G, Avsec Z̆, Gagneur J, Theis FJ. Deep learning: new computational modelling techniques for genomics. Nat Rev Genet. 2019;20(7):389–403. 10.1038/s41576-019-0122-6 30971806

[65] Zou J, Huss M, Abid A, Mohammadi P, Torkamani A, Telenti A. A primer on deep learning in genomics. Nat Genet. 2019;51(1):12–8. 10.1038/s41588-018-0295-5. 10.1038/s41588-018-0295-5 PMC11180539 30478442

[66] Kingma DP, Ba JL. Adam: A method for stochastic optimization. 2014. arXiv preprint arXiv:1412.6980. https://arxiv.org/pdf/1412.6980.pdf.

[67] Ruder S. An overview of gradient descent optimization algorithms. 2016. arXiv preprint arXiv:1609.04747.

[68] Breheny P. The group exponential lasso for bi‐level variable selection. Biometrics. 2015;71(3):731-40. 10.1111/biom.12300 25773593

[69] Endelman JB. Ridge regression and other kernels for genomic selection with R package rrBLUP. Plant Genome. 2011;4(3):250–55.

[70] Friedman J (2001). Greedy function approximation: a gradient boosting machine. Ann Stat..

[71] Friedman J, Hastie T, Tibshirani R, Narasimhan B, Tay K, Simon N, Qian J. Package ‘glmnet’. J Stat Softw. 2022;2010a:33(1).

[72] Greenwell B, Boehmke B, Cunningham J. Package ‘gbm’. R package version. 2019;2(5).

[73] Dimitriadou E, Hornik K, Leisch F, Meyer D, Weingessel A. "Package ‘e1071’." R Software package. 2009. Avaliable at https://cran.r-project.org/web/packages/e1071/index.html.

[74] Agrawal A (2019). TensorFlow Eager: A multi-stage, Python-embedded DSL for machine learning. Proc Mach Learn Syst..

[75] McKinney W. Python for data analysis: Data wrangling with Pandas, NumPy, and IPython. California: O’Reilly Media, Inc.; 2012.

