# Supplementary material for: Genomic prediction using machine learning: a comparison of the performance of regularized regression, ensemble, instance-based and deep learning methods on synthetic and empirical data
Source: BMC Genomics. 2024 Feb 7;25:152. doi: 10.1186/s12864-023-09933-x (PMC10848392; doi:10.1186/s12864-023-09933-x)
Supplement: Supplementary file 4 — Additional file 4. Includes SAS code for (i) the phenotypic data analysis (S1 Text.doc); (ii) SNP grouping schemes (S2 Text.doc); and (iii) the 5-fold data split (S3 Text.doc & S4 Text.doc) for the KWS $2010-2012$2010-2012 data sets. [file 12864_2023_9933_MOESM4_ESM.zip › S1Text.docx]

/*----SAS code for performing phenotypic data analysis for the KWS 2010, 2011 and 2012 data sets. The adjusted means (Least square means) from this code are used as the response variable for genomic prediction.****/

**PROC** **IMPORT** OUT= WORK.GENO_1

DATAFILE= "E:\Joseph2013\Synbreed_2010_2013\data\geno_trans_1.txt"

DBMS=DLM REPLACE;

DELIMITER='20'x;

GETNAMES=YES;

DATAROW=**2**;

GUESSINGROWS=**2000**;

**RUN**;

**PROC** **IMPORT** OUT= WORK.GENO_2

DATAFILE= "E:\Joseph2013\Synbreed_2010_2013\data\geno_trans_2.txt"

DBMS=DLM REPLACE;

DELIMITER='20'x;

GETNAMES=YES;

DATAROW=**2**;

GUESSINGROWS=**2000**;

**RUN**;

**PROC** **IMPORT** OUT= WORK.COVAR

DATAFILE= "E:\Joseph2013\Synbreed_2010_2013\data\covar.txt"

DBMS=TAB REPLACE;

GETNAMES=YES;

DATAROW=**2**;

**RUN**;

**PROC** **IMPORT** OUT= WORK.MAP

DATAFILE= "E:\Joseph2013\Synbreed_2010_2013\data\map .txt"

DBMS=TAB REPLACE;

GETNAMES=YES;

DATAROW=**2**;

GUESSINGROWS=**2000**;

**RUN**;

**PROC** **IMPORT** OUT= WORK.pedigree

DATAFILE= "E:\Joseph2013\Synbreed_2010_2013\data\pedigree .txt"

DBMS=DLM REPLACE;

DELIMITER='20'x;

GETNAMES=YES;

DATAROW=**2**;

GUESSINGROWS=**10000**;

**RUN**;

libname KWS 'E:\Joseph2013\Synbreed_2010_2013\Results';

**proc** **sort** data=covar;

by id;

**run**;

**proc** **sort** data=geno_1;

by SNPS;

**run**;

**Proc** **sort** data=geno_2;

by SNPS;

**run**;

**data** geno;

merge geno_1 geno_2;

by SNPS;

**run**;

**proc** **transpose** data=geno out=geno2(rename=(_name_=id)drop=_label_) prefix=Z;

**run**;

**proc** **sort** data=geno2;

by id;

**run**;

**proc** **sort** data=covar;

by id;

**run**;

**proc** **sort** data=pedigree;

by id;

**run**;

**data** geno3;

merge covar pedigree geno2;

by id;

**run**;

**data** geno4;set geno3;

array xx Z1-Z32217;

do over xx;

xx=xx-**1**;

/*if xx=. then index=1;

else Index=0;*/

end;

**run**;

**PROC** **IMPORT** OUT= WORK.Phenotypes2010

DATAFILE= "E:\Joseph_2009\Bionformatics\Synbreed\KWS\data\SynbreedA2_phenotypicData_outlierCorrected_14052012.xlsx"

DBMS=EXCEL REPLACE;

RANGE="SynbreedA2_phenotypicData_outli$";

GETNAMES=YES;

MIXED=NO;

SCANTEXT=YES;

USEDATE=YES;

SCANTIME=YES;

**RUN**;

**PROC** **IMPORT** OUT= WORK.Phenotypes2011

DATAFILE= "E:\Joseph2013\Synbreed_2010_2013\data\Phenotypes2011.xlsx"

DBMS=EXCEL REPLACE;

RANGE="Phenotypes2011$";

GETNAMES=YES;

MIXED=NO;

SCANTEXT=YES;

USEDATE=YES;

SCANTIME=YES;

**RUN**;

**PROC** **IMPORT** OUT= WORK.Phenotypes2012

DATAFILE= "E:\Joseph2013\Synbreed_2010_2013\data\Phenotypes2012.xlsx"

DBMS=EXCEL REPLACE;

RANGE="Phenotypes2012$";

GETNAMES=YES;

MIXED=NO;

SCANTEXT=YES;

USEDATE=YES;

SCANTIME=YES;

**RUN**;

/*------Preparing the full data set----------*/;

**data** phenotypes2011_2012; set phenotypes2011 phenotypes2012;

column2=input(column, **8.**);

band2 =input(band, **8.**);

drop column band ;

rename column2=column band2=band;

**run**;

**Data** phenotypes;

set phenotypes2010 phenotypes2011_2012;

subgroup2=input(subgroup, **8.**);

drop column band subgroup;

rename subgroup2=subgroup;

**run**;

**proc** **sort** data=geno4(rename=(id=labcode));

by year labcode;

**run**;

**proc** **sort** data=phenotypes;

by year labcode;

**run**;

**data** Geno_pheno;

merge Phenotypes(in=p) geno4(in=m);

by year labcode;

if p=**1** and m=**1** then source='both ';

if p=**1** and m=**0** then source='pheno';

if p=**0** and m=**1** then source='marker';

**run**;

/*----Preparing the gamma matrix for each year-----**/

**data** markers_2010 markers_2011 markers_2012 ;

set geno_pheno;

if source='both';

if year=**2010** then output markers_2010;

if year=**2011** then output markers_2011;

if year=**2012** then output markers_2012;

*keep Year Z:;

**run**;

/*------Number of genotyped genotypes in 2010=1073----*/;

**proc** **sort** data=markers_2010 out=gamma2010_1(keep=Z1-Z32217) nodupkey;

by labcode;

**run**;

**Proc** **iml**;

use gamma2010_1;

read all var _num_ into Z_2010;

gamma_2010_2=Z_2010*t(Z_2010);

create gamma_2010_2 from gamma_2010_2;

append from gamma_2010_2;

**quit**;

**data** gamma_2010;

set gamma_2010_2;

parm=**1**;

row=_n_;

**run**;

/*------Number of genotyped genotypes in 2011=857----*/;

**proc** **sort** data=markers_2011 out=gamma2011_1(keep=Z1-Z32217) nodupkey;

by labcode;

**run**;

**Proc** **iml**;

use gamma2011_1;

read all var _num_ into Z_2011;

gamma_2011_2=Z_2011*t(Z_2011);

create gamma_2011_2 from gamma_2011_2;

append from gamma_2011_2;

**quit**;

**data** gamma_2011;

set gamma_2011_2;

parm=**1**;

row=_n_;

**run**;

/*------Number of genotyped genotypes in 2012=1085----*/;

**proc** **sort** data=markers_2012 out=gamma2012_1(keep=Z1-Z32217) nodupkey;

by labcode;

**run**;

**Proc** **iml**;

use gamma2012_1;

read all var _num_ into Z_2012;

gamma_2012_2=Z_2012*t(Z_2012);

create gamma_2012_2 from gamma_2012_2;

append from gamma_2012_2;

**quit**;

**data** gamma_2012;

set gamma_2012_2;

parm=**1**;

row=_n_;

**run**;

/*-----Preparing phenotypic data for 2010 for analysis----------------------*/;

**data** phenotypes2010_2; set Geno_pheno;

where year=**2010**;

/*----Recodong testers------*/;

if tester='T1' then Tester2='T1';

else if tester='T2' then Tester2='T2';

else Tester2='T0';

/*-----Defining groups: GRP---*/

If labcode in('C1','C2','C3','C4','C5', 'C5','C6') then GRP=labcode;

if Tester='fT' then GRP='fT';

if Tester='' and labcode ^in('C1','C2','C3','C4','C5', 'C5','C6') and genotyped ne 'TRUE' then GRP='nT';

if tester='T1' and genotyped ne 'TRUE' then GRP='G0';

if tester='T2' and genotyped ne 'TRUE' then GRP='G0';

if tester='T1' and genotyped='TRUE' and subgroup=**1** then GRP='G1';

if tester='T1' and genotyped='TRUE' and subgroup=**3** then GRP='G3';

if tester='T2' and genotyped='TRUE' and subgroup=**2** then GRP='G2';

if tester='T2' and genotyped='TRUE' and subgroup=**3** then GRP='G3';

/*-----Defining SWITCH1------*/

if GRP in ('G1', 'G2','G3') then SWITCH1= **1**;

else SWITCH1=**0**;

/*----Defining SWITCH2---*/;

if GRP in ('G0', 'fT','nT') then SWITCH2=**1**;

else SWITCH2=**0**;

/*-Grouping genotypes in terms of genotyped vs nongenotyped lines---*/;

If GRP in ('G1','G2','G3') then G2=labcode;

else G2='1 ';

/*-Grouping genotypes in terms of genotyped vs nongenotyped lines--*/;

If G2 in ('1') then G3='MA10411_00001'; *Needed to ensure that Switch2*G3=698=dimension of ldata (i..e Z*t(Z));

else G3=G2; ;

/*-----Defining groups: G-----*/;

G=labcode;

drop Z:;

**run**;

/*-----Preparing phenotypic data for 2011 for analysis------------------------*/;

**data** phenotypes2011_2; set Geno_pheno;

where year=**2011**;

/*----Recodong testers------*/;

if tester='T1' then Tester2='T1';

else if tester='T3' then Tester2='T3';

else if tester='' then Tester2='T0';

/*-----Defining groups: GRP---*/

If labcode in('C2','C3','C5', 'C7','C8') then GRP=labcode;

*if Tester='fT' then GRP='fT';

if Tester='' and labcode ^in('C1','C2','C3','C4','C5', 'C5','C6') and genotyped ne 'TRUE' then GRP='nT';

if tester='T1' and genotyped ne 'TRUE' then GRP='G0';

*if tester='T2' and genotyped ne 'TRUE' then GRP='G0';

if tester='T3' and genotyped ne 'TRUE' then GRP='G0';

if tester='T1' and genotyped='TRUE' and subgroup=**1** then GRP='G1';

if tester='T1' and genotyped='TRUE' and subgroup=**3** then GRP='G3';

if tester='T3' and genotyped='TRUE' and subgroup=**1** then GRP='G1';

if tester='T3' and genotyped='TRUE' and subgroup=**2** then GRP='G2';

if tester='T3' and genotyped='TRUE' and subgroup=**3** then GRP='G3';

/*-----Defining SWITCH1------*/

if GRP in ('G1', 'G2','G3') then SWITCH1= **1**;

else SWITCH1=**0**;

/*----Defining SWITCH2---*/;

if GRP in ('G0', 'nT') then SWITCH2=**1**;

else SWITCH2=**0**;

/*-grouping genotypes in terms of genotyped vs non-genotyped lines-*/;

If GRP in ('G1','G2','G3') then G2=labcode;

else G2='1 ';

/*-----grouping genotypes in terms of genotyped vs nongenotyped lines---*/;

If G2 in ('1') then G3='MA11405_00001'; *Needed to ensure that Switch3*G3=698=dimension of ldata (i..e Z*t(Z));

else G3=G2; ;

/*-----Defining groups: G-----*/;

G=labcode;

drop Z:;

**run**;

/*-----Preparing phenotypic data for 2012 for analysis------------------------*/;

**data** phenotypes2012_2; set Geno_pheno;

where year=**2012**;

/*----Recodong testers------*/;

if tester='T1' then Tester2='T1';

else if tester='T6' then Tester2='T6';

else if tester='T9' then Tester2='T9';

else Tester2='T0';

/*-----Defining groups: GRP---*/

If labcode in('C3','C5','C9','C10','C11','C12','C13') then GRP=labcode;

if Tester='fT' then GRP='fT';

if Tester='' and labcode ^in('C3','C5','C9','C10','C11','C12','C13') and genotyped ne 'TRUE' then GRP='nT';

if tester='T1' and genotyped ne 'TRUE' then GRP='G0';

if tester='T6' and genotyped ne 'TRUE' then GRP='G0';

if tester='T9' and genotyped ne 'TRUE' then GRP='G0';

if tester='T1' and genotyped='TRUE' and subgroup=**1** then GRP='G1';

if tester='T1' and genotyped='TRUE' and subgroup=**3** then GRP='G3';

if tester='T6' and genotyped='TRUE' and subgroup=**1** then GRP='G1';

if tester='T6' and genotyped='TRUE' and subgroup=**3** then GRP='G3';

if tester='T9' and genotyped='TRUE' and subgroup=**1** then GRP='G1';

if tester='T9' and genotyped='TRUE' and subgroup=**3** then GRP='G3';

if tester='T9' and genotyped='TRUE' and subgroup=**4** then GRP='G4';

if tester='T9' and genotyped='TRUE' and subgroup=**5** then GRP='G5';

/*-----Defining SWITCH1------*/

if GRP in ('G1', 'G2','G3','G4','G5') then SWITCH1= **1**;

else SWITCH1=**0**;

/*----Defining SWITCH2---*/;

if GRP in ('G0', 'fT','nT') then SWITCH2=**1**;

else SWITCH2=**0**;

/*--Grouping genotypes in terms of genotyped vs non-genotyped lines-*/;

If GRP in ('G1','G2','G3','G4','G5') then G2=labcode;

else G2='1 ';

/*--Grouping genotypes in terms of genotyped vs nongenotyped lines---*/;

If G2 in ('1') then G3='MA10411_00334'; *Needed to ensure that Switch3*G3=698=dimension of ldata (i..e Z*t(Z));

else G3=G2; ;

/*-----Defining groups: G-----*/;

G=labcode;

drop Z:;

**run**;

/*--Model assuming uncorrelated genotypes with testers: start values---*/;

**proc** **hpmixed** data=phenotypes2010_2 method=REML;

ods output covparms=covparm_sstage_tester_2010 lsmeans=lsmeans_sstage_tester_2010 ;

class LOC TRIAL REP BLOCK Tester2 GRP G G2 ;

model GDY= Tester2 GRP Tester2*GRP Tester2*GRP*G;

Random LOC LOC*TRIAL LOC*TRIAL*REP LOC*TRIAL*REP*BLOCK Tester2*GRP*SWITCH2*G Tester2*GRP*SWITCH1*G2 ;

*parms (36.4141) (20.9845)(5.3370)(9.0311) (0) (0)(187.73);

lsmeans Tester2*GRP Tester2*GRP*G;

**run**;

/*-Model assuming uncorrelated genotypes with testers: start values----*/;

**proc** **hpmixed** data=phenotypes2011_2 method=REML;

ods output covparms=covparm_sstage_tester_2011 lsmeans=lsmeans_sstage_tester_2011 ;

class LOC TRIAL REP BLOCK Tester2 GRP G G2 ;

model GDY= Tester2 GRP Tester2*GRP Tester2*GRP*G;

Random LOC LOC*TRIAL LOC*TRIAL*REP LOC*TRIAL*REP*BLOCK Tester2*GRP*SWITCH2*G Tester2*GRP*SWITCH1*G2 ;

parms (**9.5476**) (**17.5842**)(**6.8896**)(**10.0562**) (**3.9191**) (**0.02512**)(**93.0742**);

lsmeans Tester2*GRP Tester2*GRP*G;

**run**;

**proc** **hpmixed** data=phenotypes2012_2 method=REML;

ods output covparms=covparm_sstage_tester_2012 lsmeans=lsmeans_sstage_tester_2012 ;

class LOC TRIAL REP BLOCK Tester2 GRP G G2 ;

model GDY= Tester2 GRP Tester2*GRP Tester2*GRP*G;

Random LOC LOC*TRIAL LOC*TRIAL*REP LOC*TRIAL*REP*BLOCK Tester2*GRP*SWITCH2*G Tester2*GRP*SWITCH1*G2 ;

parms (**83.1231**) (**38.3938**)(**6.3658**)(**13.2492**) (**0**) (**0**)(**67.7402**);

lsmeans Tester2*GRP Tester2*GRP*G;

**run**;

/*-----Model assuming correlated genotypes: RR-BLUP----*/;

/*proc mixed data=phenotypes2010_2 maxiter=1000 maxfunc=5000 ;

ods output covparms=covparms_corr_tester_2010 solutionR=solR_tester_2010;

class LOC TRIAL REP BLOCK Tester2 GRP G G3;

model GDY= Tester2 GRP ;

Random SWITCH1*G3/ldata=gamma_2010 type=lin(1);

Random LOC LOC*TRIAL LOC*TRIAL*REP LOC*TRIAL*REP*BLOCK Tester2*GRP*SWITCH2*G/s ;

parms (0.006185) (36.3978) (20.9752)(5.3364)(9.0266) (0) (187.73);

run;

proc mixed data=phenotypes2011_2 maxiter=1000 maxfunc=5000 ;

ods output covparms=covparms_corr_tester_2011 solutionR=solR_tester_2011;

class LOC TRIAL REP BLOCK Tester2 GRP G G3;

model GDY= Tester2 GRP;

Random SWITCH1*G3/ldata=gamma_2011 type=lin(1);

Random LOC LOC*TRIAL LOC*TRIAL*REP LOC*TRIAL*REP*BLOCK Tester2*GRP*SWITCH2*G/s ;

parms (0.006185) (9.3857) (17.0100)(6.9364)(8.7360) (47.0877) (96.1601);

run;*/

/*-----Model assuming correlated genotypes: for estimating variance components----*/;

/*proc mixed data=phenotypes2012_2 maxiter=1000 maxfunc=5000 ;

ods output covparms=covparms_corr_tester_2012 solutionR=solR_tester_2012;

class LOC TRIAL REP BLOCK Tester2 GRP G G3;

model GDY= Tester2 GRP ;

Random SWITCH1*G3/ldata=gamma_2012 type=lin(1);

Random LOC LOC*TRIAL LOC*TRIAL*REP LOC*TRIAL*REP*BLOCK Tester2*GRP*SWITCH2*G/s ;

*parms (0.005892) (0.8148) (0.9579)(0)(26.4686) (124.62);

run;

/*----Creating a data set with lsmeans and markers for each year---------*/;

**proc** **sort** data=markers_2010 out=markers_2010_a(keep=labcode Z:) nodupkey;

by labcode;

**run**;

**proc** **sort** data=markers_2011 out=markers_2011_a(keep=labcode Z:) nodupkey;

by labcode;

**run**;

**proc** **sort** data=markers_2012 out=markers_2012_a(keep=labcode Z:) nodupkey;

by labcode;

**run**;

**proc** **sort** data=lsmeans_sstage_tester_2010;

by G;

**run**;

**proc** **sort** data=lsmeans_sstage_tester_2011;

by G;

**run**;

**proc** **sort** data=lsmeans_sstage_tester_2012;

by G;

**run**;

**data** geno_pheno_2010;

merge lsmeans_sstage_tester_2010(rename=(G=labcode)) markers_2010_a;

by labcode;

if Z1 ^=**.**;

drop effect Tester2 GRP Stderr DF tValue Probt;

**run**;

**data** geno_pheno_2011;

merge lsmeans_sstage_tester_2011(rename=(G=labcode)) markers_2011_a;

by labcode;

if Z1 ^=**.**;

drop effect Tester2 GRP Stderr DF tValue Probt;

**run**;

**data** geno_pheno_2012;

merge lsmeans_sstage_tester_2012(rename=(G=labcode)) markers_2012_a;

by labcode;

if Z1 ^=**.**;

drop effect Tester2 GRP Stderr DF tValue Probt;

**run**;

/*---Computing the effects of Tester2*GRP: Only Known testers are considered -*/;

**Proc** **means** data=lsmeans_sstage_tester_2010 (where=(Tester2 in ('T1', 'T2') and GRP in ('G1','G2','G3') and G='')) nway noprint;

var Estimate;

output out=Tester_GRP_2010 (drop=_type_ _freq_) mean=;

**run**;

**data** Tester_GRP_2010_2; set lsmeans_sstage_tester_2010;

Effect=Estimate-**124.83**;

Keep Effect Tester2 GRP;

**run**;

**Proc** **means** data=lsmeans_sstage_tester_2011 (where=(Tester2 in ('T1', 'T2') and GRP in ('G1','G2','G3') and G='')) nway noprint;

var Estimate;

output out=Tester_GRP_2011 (drop=_type_ _freq_) mean=;

**run**;

**data** Tester_GRP_2011_2; set lsmeans_sstage_tester_2011;

Effect=Estimate-**140.58**;

Keep Effect Tester2 GRP;

**run**;

**Proc** **means** data=lsmeans_sstage_tester_2012 (where=(Tester2 in ('T1', 'T6', 'T9') and GRP in ('G1','G2','G3') and G='')) nway noprint;

var Estimate;

output out=Tester_GRP_2012 (drop=_type_ _freq_) mean=;

**run**;

**data** Tester_GRP_2012_2; set lsmeans_sstage_tester_2012;

Effect=Estimate-**143.01**;

Keep Effect Tester2 GRP;

**run**;
