# Supplementary material for: Genomic prediction using machine learning: a comparison of the performance of regularized regression, ensemble, instance-based and deep learning methods on synthetic and empirical data
Source: BMC Genomics. 2024 Feb 7;25:152. doi: 10.1186/s12864-023-09933-x (PMC10848392; doi:10.1186/s12864-023-09933-x)
Supplement: Supplementary file 4 — Additional file 4. Includes SAS code for (i) the phenotypic data analysis (S1 Text.doc); (ii) SNP grouping schemes (S2 Text.doc); and (iii) the 5-fold data split (S3 Text.doc & S4 Text.doc) for the KWS $2010-2012$2010-2012 data sets. [file 12864_2023_9933_MOESM4_ESM.zip › S2Text.docx]

/*-----SAS Macro for grouping SNP markers into groups of sizes 10,20,…100 separately for each of the 5 validation sets in each of the 10 replicates. This example code is for Validation set 1 in the KWS 2010 data set. Similar macros are used for validation sets 2 to 4 for the KWS 2010 data set. This is then repeated for the KWS 2011 and 2012 data sets for a total of 15 macros, 5 for each KWS 2010, 2011 and 2012.*/

*proc printto log="D:\Joseph2013\Synbreed_2010_2013\Results\log4.tst" new; **run**;

/*-----------Creating groups of markers--------------------------------------*/;

**PROC** **IMPORT** OUT= WORK.map(rename=(SNPS=SNPName))

DATAFILE= "D:\Joseph2013\Synbreed_2010_2013\data\map .txt"

DBMS=TAB REPLACE;

GETNAMES=YES;

DATAROW=**2**;

GUESSINGROWS=**2000**;

**RUN**;

**data** map2; set map;

SNP=_N_;

**run**;

*options mprint mlogic symbolgen;

**%macro** ***Waraper***;

%do k=**1** %to **10**; *--------Indexes the 10 files to be imported;

%do v=**1** %to **1**; *--------Indexes the 5 validation sets to be imported---*/;

/*---Import file containing markers with zero variance------*/;

PROC IMPORT OUT= WORK.ZEROVARIANCE (rename=(x=markers) drop=Var2)

DATAFILE= "D:\Joseph2013\Synbreed_2010_2013\data\Markers_with_zero_var_2010_Rep&k._v&v..txt"

DBMS=TAB REPLACE;

GETNAMES=YES;

DATAROW=**2**;

GUESSINGROWS=**2000**;

RUN;

data zerovariance2; set zerovariance;

markers2=compress(markers,'Z'); *drop Z from marker names;

drop markers;

SNP=input(markers2, **8.**);

run;

Proc sort data=map2;

by SNP;

run;

proc sort data=ZEROVARIANCE2;

by SNP;

run;

/*------markers with zero variances are deleted prior to analysis------*/;

data map3;

merge map2 ZEROVARIANCE2(in=x2);

by SNP;

if x2=**1** then index=**1**;

if index ne **1**;

keep SNP SNPname Chr Pos;

run;

data map3; set map3; /**Renumber remaining markers---*/;

SNP=_N_;

run;

proc means data=map3 nway noprint;

class Chr; *chromosome;

var SNP;

output out=map4(drop=_type_ _freq_) n=;

run;

proc sort data=Map3;

by SNP;

run;

*Assign values of n1 to n10 in the data set map4 to macro variables n1 to n10;

data _null_;

set map4;

if chr=**1** then call symput('n1',SNP);

else if chr=**2** then call symput('n2',SNP) ;

else if chr=**3** then call symput('n3',SNP) ;

else if chr=**4** then call symput('n4',SNP) ;

else if chr=**5** then call symput('n5',SNP) ;

else if chr=**6** then call symput('n6',SNP) ;

else if chr=**7** then call symput('n7',SNP) ;

else if chr=**8** then call symput('n8',SNP) ;

else if chr=**9** then call symput('n9',SNP) ;

else call symput('n10',SNP) ;

run;

**%macro** groups (n1, n2, n3, n4, n5, n6, n7, n8, n9, n10);

%do g=**10** %to **100** %by **10**;

data Chromosome1;

do index=**1** to ceil(%sysevalf((&n1/&g)));

do j= **1** to &g;

output;

end;

end;

run;

data Chromosome_1; set Chromosome1;

SNP=_N_;

if SNP<=&n1;

run;

data Chromosome2;

do index=**1** to ceil(%sysevalf((&n2/&g)));

do j= **1** to &g;

output;

end;

end;

run;

data Chromosome_2; set Chromosome2;

SNP=_N_;

if SNP<=&n2;

Index=Index+ceil(%sysevalf((&n1/&g)));

run;

data Chromosome3;

do index=**1** to ceil(%sysevalf((&n3/&g)));

do j= **1** to &g;

output;

end;

end;

run;

data Chromosome_3; set Chromosome3;

SNP=_N_;

if SNP<=&n3;

Index=Index+ceil(%sysevalf((&n1/&g)))+ceil(%sysevalf((&n2/&g)));

run;

data Chromosome4;

do index=**1** to ceil(%sysevalf((&n4/&g)));

do j= **1** to &g;

output;

end;

end;

run;

data Chromosome_4; set Chromosome4;

SNP=_N_;

if SNP<=&n4;

Index=Index+ceil(%sysevalf((&n1/&g)))+ceil(%sysevalf((&n2/&g)))+ceil(%sysevalf((&n3/&g)));

run;

data Chromosome5;

do index=**1** to ceil(%sysevalf((&n5/&g)));

do j= **1** to &g;

output;

end;

end;

run;

data Chromosome_5; set Chromosome5;

SNP=_N_;

if SNP<=&n5;

Index=Index+ceil(%sysevalf((&n1/&g)))+ceil(%sysevalf((&n2/&g)))+ceil(%sysevalf((&n3/&g)))

+ceil(%sysevalf((&n4/&g)));

run;

data Chromosome6;

do index=**1** to ceil(%sysevalf((&n6/&g)));

do j= **1** to &g;

output;

end;

end;

run;

data Chromosome_6; set Chromosome6;

SNP=_N_;

if SNP<=&n6;

Index=Index+ceil(%sysevalf((&n1/&g)))+ ceil(%sysevalf((&n2/&g)))+ceil(%sysevalf((&n3/&g)))

+ceil(%sysevalf((&n4/&g)))+ceil(%sysevalf((&n5/&g)));

run;

data Chromosome7;

do index=**1** to ceil(%sysevalf((&n7/&g)));

do j= **1** to &g;

output;

end;

end;

run;

data Chromosome_7; set Chromosome7;

SNP=_N_;

if SNP<=&n7;

Index=Index+ceil(%sysevalf((&n1/&g)))+ceil(%sysevalf((&n2/&g)))+ceil(%sysevalf((&n3/&g)))

+ceil(%sysevalf((&n4/&g)))+ceil(%sysevalf((&n5/&g)))+ceil(%sysevalf((&n6/&g)));

run;

data Chromosome8;

do index=**1** to ceil(%sysevalf((&n8/&g)));

do j= **1** to &g;

output;

end;

end;

run;

data Chromosome_8; set Chromosome8;

SNP=_N_;

if SNP<=&n8;

Index=Index+ceil(%sysevalf((&n1/&g)))+ceil(%sysevalf((&n2/&g)))+ceil(%sysevalf((&n3/&g)))

+ceil(%sysevalf((&n4/&g)))+ceil(%sysevalf((&n5/&g)))+ceil(%sysevalf((&n6/&g)))

+ceil(%sysevalf((&n7/&g)));

run;

data Chromosome9;

do index=**1** to ceil(%sysevalf((&n9/&g)));

do j= **1** to &g;

output;

end;

end;

run;

data Chromosome_9; set Chromosome9;

SNP=_N_;

if SNP<=&n9;

Index=Index+ceil(%sysevalf((&n1/&g)))+ceil(%sysevalf((&n2/&g)))+ceil(%sysevalf((&n3/&g)))

+ceil(%sysevalf((&n4/&g)))+ceil(%sysevalf((&n5/&g)))+ceil(%sysevalf((&n6/&g)))

+ceil(%sysevalf((&n7/&g)))+ceil(%sysevalf((&n8/&g)));

run;

data Chromosome10;

do index=**1** to ceil(%sysevalf((&n10/&g)));

do j= **1** to &g;

output;

end;

end;

run;

data Chromosome_10; set Chromosome10;

SNP=_N_;

if SNP<=&n10;

Index=Index+ceil(%sysevalf((&n1/&g)))+ceil(%sysevalf((&n2/&g)))+ceil(%sysevalf((&n3/&g)))

+ceil(%sysevalf((&n4/&g)))+ceil(%sysevalf((&n5/&g)))+ceil(%sysevalf((&n6/&g)))

+ceil(%sysevalf((&n7/&g)))+ceil(%sysevalf((&n8/&g)))+ceil(%sysevalf((&n9/&g)));

run;

Data index_&g;

set chromosome_1-Chromosome_10;

rename Index=Index&g;

keep Index SNP;

run;

data Index_&g; set Index_&g;

SNP=_N_;

run;

proc sort data=Index_&g; by SNP; run;

%end;

**%mend**;

%***groups***(n1=&n1, n2=&n2, n3=&n3, n4=&n4, n5=&n5, n6=&n6, n7=&n7, n8=&n8, n9=&n9, n10=&n10);

data Groups_2010_Rep&k._v&v;

*----Reorder the position of the variables in the datafile----*/;

Retain SNPName chr pos SNP Replicate Fold Index1 Index10 Index20 Index30 Index40 Index50 Index60 Index70 Index80 Index90 Index100;

merge map3 Index_:;

by SNP;

Replicate=&k;

Index1=**1**;

Fold=&v;

run;

Proc append data=Groups_2010_Rep&k._v&v base=Groups_2010_Reps_v1; run;

/*---Groups of sizes 10,20,..., 1000 had respectively 1000, 500, 335,250, 200, 170, 145, 125, 115 and 100 SNPs.***/;

%end;

%end;

proc datasets; delete Groups_2010_Rep&k._v&v; run; quit;

**%mend**;

%***Waraper***;

**proc** **sort** data=Groups_2010_Reps_v1; by replicate fold chr SNP; **run**;

**PROC** **EXPORT** DATA= WORK.Groups_2010_Reps_v1

OUTFILE= "D:\Joseph2013\Synbreed_2010_2013\data\groups_2010_Reps_v1.txt"

DBMS=TAB REPLACE;

PUTNAMES=YES;

**RUN**;

/*-------*Check the bottom of file for each replicate and fold to be sure last and second last obs are identical.

if they are not then make the last entry equal to the second last entry---**/
