# Supplementary material for: Genomic prediction using machine learning: a comparison of the performance of regularized regression, ensemble, instance-based and deep learning methods on synthetic and empirical data
Source: BMC Genomics. 2024 Feb 7;25:152. doi: 10.1186/s12864-023-09933-x (PMC10848392; doi:10.1186/s12864-023-09933-x)
Supplement: Supplementary file 4 — Additional file 4. Includes SAS code for (i) the phenotypic data analysis (S1 Text.doc); (ii) SNP grouping schemes (S2 Text.doc); and (iii) the 5-fold data split (S3 Text.doc & S4 Text.doc) for the KWS $2010-2012$2010-2012 data sets. [file 12864_2023_9933_MOESM4_ESM.zip › S3Text.docx]

/***********************************************************************/

Xie’s macro used to split KWS 2010, 2011 and 2012 data sets into 5 parts based on a specified probability vector

/************************************************************************/

**%macro** split(

seed, /*seed for random number generator */

probvector, /*macro variable of splitting prob vector separated by " " */

control_vars, /*control variable as in PROC SURVEYSELECT */

in_dsn, /*input data set */

out_dsn=_out_dsn_,

sort=nest

);

%let blank=%str( );

%let n=1; %let nv=1;

%let probs=&probvector;

%let dsid=%sysfunc(open(&in_dsn));

%if (&dsid=**0**) %then %do;

%put %sysfunc(sysmsg());

%put Stop Processing due to Errors;

%goto exit;

%end;

%else %do;

%let nobs=%sysfunc(attrn(&dsid, NLOBS));

%let dsid=%sysfunc(close(&dsid));

%end;

%if &out_dsn=&blank %then %let &out_dsn=&in_dsn;

%if &seed=&blank %then %let seed=0;

%let pos=%scan(&probs,&n,&blank);

%do %while(&pos ne &blank);

%let n=%eval(&n+1);

%let pos=%scan(&probs,&n,&blank);

%end;

%let n=%eval(&n-1);

%let pos=%scan(&control_vars,&nv,&blank);

%let control_vars_x=&pos;

%let control_key=%str(&pos);

%let control_find=%str(key:&pos);

%do %while(&pos ne &blank);

%put &pos;

%let nv=%eval(&nv+1);

%let pos=%scan(&control_vars,&nv,&blank);

%if &pos^=&blank %then %do;

%let control_vars_x=%str(&control_vars_x * &pos);

%let control_key=%str(&pos%", %"&control_key);

%let control_find=%str(key:&pos, &control_find);

%end;

%end;

%let nv=%eval(&nv-1);

%put nv=&nv;

%if %eval(&nv>**0**) %then %do;

%if %upcase(&sort)=NEST %then %do;

proc sort data=&in_dsn out=_in_dsn_;

by &control_vars;

run;

%end;

%else %if %upcase(&sort)=SERP %then %do;

ods select none;

proc surveyselect data=&in_dsn samprate=**1.0** out=_in_dsn_

sort=&sort method=sys;

control &control_vars;

run;

ods select all;

%end;

%else %do;

%put Specify only NEST or SERP for sort statement;

%goto exit;

%end;

%let in_dsn=_in_dsn_;

proc freq data=_in_dsn_ noprint;

tables &control_vars_x /missing out=_freq_;

run;

proc sql noprint;

select name into :keyvars separated by ' '

from sashelp.vcolumn

where libname='WORK' &

upcase(memname)='_FREQ_' &

name not in ('COUNT', 'PERCENT')

;

quit;

data _freq_(index=(idx=(&keyvars)));

set _freq_ nobs=ntotal end=eof;

_index_=_n_;

if eof then call symput('ncomb', ntotal);

run;

%let withcontrol=1;

%end;

%else %do;

%let withcontrol=0;

%let ncomb=1;

%end;

proc sql noprint;

select name into :originalvars separated by ' '

from sashelp.vcolumn

where libname='WORK' &

upcase(memname)='_IN_DSN_'

;

quit;

data probs;

%do i=**1** %to &n;

_prob_&i=%scan(&probs, &i, &blank);

%end;

output;

drop i;

run;

%let i=&n;

%put Initial counts: n=&n i=&i ncomb=&ncomb;

data &out_dsn _N;

array _P{*} _prob_1-_prob_&n;

array _N{**0**:%eval(&n+**1**)} N_0-N_%eval(&n+**1**);

array _Pmat{**1**:&ncomb, **1**:&n} _temporary_;

array _Fmat{**1**:&ncomb, **1**:&n} _temporary_;

array _Xmat{**1**:&ncomb, **1**:&n} _temporary_;

array _Kmat{**1**:&ncomb, **1**:&n} _temporary_;

array _Nmat{**1**:&ncomb, **0**:%eval(&n+**1**)} _temporary_;

if _n_=**1** then do;

set probs;

do i=**1** to &ncomb;

do j=**1** to &n;

_Pmat[i, j]=_P[j];

if j=**1** then _Fmat[i, j]=_P[j];

else _Fmat[i, j]=_Fmat[i, j-**1**]+_P[j];

end;

do j=**1** to &n;

_Fmat[i, j]=_Fmat[i, j]/_Fmat[i, &n];

end;

end;

put 'FINISH LOADING PROB VECTORS';

%if &withcontrol=**1** %then %do;

do while (^eof);

set _freq_ end=eof;

cn=**0**;

_Nmat[_index_, **0**]=COUNT; _Nmat[_index_, %eval(&n+**1**)]=**0**;

do j=**1** to &n;

if j<&n then do;

_Nmat[_index_, j]=round(_P[j]*COUNT, **1**);

cn+_Nmat[_index_, j];

end;

else _Nmat[_index_, j]=COUNT-cn;

_Xmat[_index_, j]=_Nmat[_index_, j];

_Kmat[_index_, j]=**0**;

end;

end;

%end;

%else %do;

_index_=**1**; cn=**0**;

do j=**1** to &n;

if j<&n then do;

_Nmat[_index_, j]=round(_P[j]*&nobs, **1**);

cn+_Nmat[_index_, j];

end;

else _Nmat[_index_, j]=&nobs-cn;

_Xmat[_index_, j]=_Nmat[_index_, j];

_Kmat[_index_, j]=**0**;

end;

%end;

put 'FINISH LOADING ALL DATA SETS';

do i=**1** to &ncomb;

do k=**0** to %eval(&n+**1**);

_N[k]=_Nmat[i, k]; put _Nmat[i, k]=;

end;

output _N;

end;

end;

set &in_dsn nobs=ntotal end=eof_in;

r=ranuni(&seed);

%if &withcontrol=**1** %then %do;

do until (_iorc_=%***sysrc***(_dsenom));

set _freq_ key=idx;

if _iorc_^=%***sysrc***(_sok) then _error_=**0**;

end;

%end;

%else %do;

_index_=**1**;

%end;

notfound=**1**; j=**1**;

do j=**1** to &n;

if notfound then do;

if r<=_Fmat[_index_, j] & _Kmat[_index_, j]<_Nmat[_index_,j] then do;

_BLOCK=j; notfound=**0**; _Kmat[_index_, _BLOCK]+**1**;

end;

end;

end;

if notfound then do; _BLOCK=&n; notfound=**0**; _Kmat[_index_, _BLOCK]+**1**; end;

_Xmat[_index_, _BLOCK]=_Nmat[_index_, _BLOCK]-_Kmat[_index_, _BLOCK];

_Nmat[_index_, %eval(&n+**1**)]+**1**;

if ^(eof_in ) then do;

_temp_=(_Nmat[_index_, **0**]-_Nmat[_index_, %eval(&n+**1**)]);

if _temp_<=**0** then _Pmat[_index_, _BLOCK]=**0**;

else _Pmat[_index_, _BLOCK]=_Xmat[_index_, _BLOCK]/_temp_;

do j=**1** to &n;

if j=**1** then _Fmat[_index_, j]=_Pmat[_index_, j];

else _Fmat[_index_, j]=_Fmat[_index_, j-**1**]+_Pmat[_index_, j];

end;

do j=**1** to &n;

if _Fmat[_index_, &n]<=**0** then _Fmat[_index_, j]=**0**;

else _Fmat[_index_, j]=_Fmat[_index_, j]/_Fmat[_index_, &n];

end;

end;

else if (eof_in) then do;

do i=**1** to &ncomb;

do k=**1** to %eval(&n);

_N[k]=_Kmat[i, k]; put _Kmat[i, k]=;

end;

output _N;

end;

end;

keep &originalvars _BLOCK;

output &out_dsn;

run;

%exit:

**%mend**;

*options mprint mlogic;

%put START TIME 1: %sysfunc(datetime(),datetime20.);

/*

data test0;

do ID=1 to 796773;

TDSP=min(3, round(ranuni(8976)*4));

if ranuni(93745)<0.3 then VS='H'; else VS='M';

output;

end;

run;

%put START TIME 2: %sysfunc(datetime(),datetime20.);

%let seed=99999;

%let probvector=0.1485905 0.1485905 0.2324283 0.2351954 0.2351954;

%let control_vars=TDSP VS;

%let in_dsn=test0;

%let out_dsn=test_out;

%let sort=NEST;

%split(&seed, &probvector, &control_vars, &in_dsn, out_dsn=&out_dsn);

*options nomprint nomlogic;

%put END TIME: %sysfunc(datetime(),datetime20.);

;;

proc freq data=&out_dsn noprint;

tables _BLOCK*TDSP*VS /missing out=_freq_out_;

run;*/
