# Supplementary material for: Genomic prediction using machine learning: a comparison of the performance of regularized regression, ensemble, instance-based and deep learning methods on synthetic and empirical data
Source: BMC Genomics. 2024 Feb 7;25:152. doi: 10.1186/s12864-023-09933-x (PMC10848392; doi:10.1186/s12864-023-09933-x)
Supplement: Supplementary file 4 — Additional file 4. Includes SAS code for (i) the phenotypic data analysis (S1 Text.doc); (ii) SNP grouping schemes (S2 Text.doc); and (iii) the 5-fold data split (S3 Text.doc & S4 Text.doc) for the KWS $2010-2012$2010-2012 data sets. [file 12864_2023_9933_MOESM4_ESM.zip › S4Text.docx]

/**-------SAS macro for splitting KWS data sets for 2010,2011 and 2012 into 5 approximately equal parts using stratified random sampling. The splitting of each data set into 5 parts is repeated or replicated 10 times. The marco that does the actual splitting is called macro_split_by_given_probs.sas and is read using the %include statement*/

%include "D:\Joseph_2009\Bionformatics\Cross validation\models\macro_split_by_given_probs.sas";

/*--------------Split KWS 2010 dataset into 5 parts using stratified random sampling------*/;

**data** Geno_pheno_2010_1; set "D:\Joseph2013\Synbreed_2010_2013\Results\geno_pheno_2010" ;

rename labcode = G;

keep Tester2 GRP labcode Estimate ;

**run**;

**%macro** ***replicate_2010***;

%put START TIME 2: %sysfunc(datetime(),datetime20.);

%do r=**1** %to **10**;

%let seed=%eval(1000+100*&r);

%let probvector=0.20 0.20 0.2 0.2 0.2;

%let control_vars=Tester2 GRP;

%let in_dsn=Geno_pheno_2010_1;

%let out_dsn=Lsmeans_2010_5_fold_rep&r;

%let sort=NEST;

%***split***(&seed, &probvector, &control_vars, &in_dsn, out_dsn=&out_dsn);

*options nomprint nomlogic;

%put END TIME: %sysfunc(datetime(),datetime20.);

;;

data Lsmeans_2010_5_fold_rep&r; set Lsmeans_2010_5_fold_rep&r;

Replicate=&r;

run;

%end;

**%mend**;

%***replicate_2010***;

**data** Lsmeans_2010_5_fold_rep; set Lsmeans_2010_5_fold_rep:; **run**;

**Proc** **sort** data=Lsmeans_2010_5_fold_rep;

by Replicate _BLOCK;

**run**;

**proc** **freq** data=Lsmeans_2010_5_fold_rep noprint;

by replicate;

tables _BLOCK*Tester2*GRP /missing out=_freq_out_2010;

**run**;

**proc** **means** data=_freq_out_2010 noprint nway;

by Replicate;

class _BLOCK;

var count percent;

output out=summary_2010(drop=_type_ _freq_) sum=;

**run**;

**data** Geno_pheno_2010_2; set "D:\Joseph2013\Synbreed_2010_2013\Results\geno_pheno_2010" ;

rename labcode = G;

**run**;

**proc** **sort** data = Geno_pheno_2010_2;

by Tester2 GRP G;

**run**;

**proc** **sort** data=Lsmeans_2010_5_fold_rep;

by Tester2 GRP G;

**run**;

**data** KWS_2010_5_fold;

merge Lsmeans_2010_5_fold_rep (in=x1) Geno_pheno_2010_2 (in=x2);

by Tester2 GRP G;

if x1=**1** and x2=**1**;

**run**;

**Proc** **sort** data=KWS_2010_5_fold;

by Replicate _Block Tester2 GRP G;

**run**;

/*proc printto log="D:\Joseph2013\Synbreed_2010_2013\Results\log8.tst" new; run;

ods listing close;*/

/*----USE file "D:\Joseph2013\Synbreed_2010_2013\Models"KWS_2010_2010_export" to export KWS_2011_5_fold*/;

/*--------------Split KWS 2011 dataset into 5 parts using stratified random sampling------*/;

**data** Geno_pheno_2011_1; set "D:\Joseph2013\Synbreed_2010_2013\Results\geno_pheno_2011" ;

rename labcode = G;

keep Tester2 GRP labcode Estimate ;

**run**;

**%macro** ***replicate_2011***;

%put START TIME 2: %sysfunc(datetime(),datetime20.);

%do r=**1** %to **10**;

%let seed=%eval(1100+100*&r);

%let probvector=0.20 0.20 0.2 0.2 0.2;

%let control_vars=Tester2 GRP;

%let in_dsn=Geno_pheno_2011_1;

%let out_dsn=Lsmeans_2011_5_fold_rep&r;

%let sort=NEST;

%***split***(&seed, &probvector, &control_vars, &in_dsn, out_dsn=&out_dsn);

*options nomprint nomlogic;

%put END TIME: %sysfunc(datetime(),datetime20.);

;;

data Lsmeans_2011_5_fold_rep&r; set Lsmeans_2011_5_fold_rep&r;

Replicate=&r;

run;

%end;

**%mend**;

%***replicate_2011***;

**data** Lsmeans_2011_5_fold_rep; set Lsmeans_2011_5_fold_rep:; **run**;

**Proc** **sort** data=Lsmeans_2011_5_fold_rep;

by Replicate _BLOCK;

**run**;

**proc** **freq** data=Lsmeans_2011_5_fold_rep noprint;

by replicate;

tables _BLOCK*Tester2*GRP /missing out=_freq_out_2011;

**run**;

**proc** **means** data=_freq_out_2011 noprint nway;

by Replicate;

class _BLOCK;

var count percent;

output out=summary_2011(drop=_type_ _freq_) sum=;

**run**;

**data** Geno_pheno_2011_2; set "D:\Joseph2013\Synbreed_2010_2013\Results\geno_pheno_2011" ;

rename labcode = G;

**run**;

**proc** **sort** data = Geno_pheno_2011_2;

by Tester2 GRP G;

**run**;

**proc** **sort** data=Lsmeans_2011_5_fold_rep;

by Tester2 GRP G;

**run**;

**data** KWS_2011_5_fold;

merge Lsmeans_2011_5_fold_rep (in=x1) Geno_pheno_2011_2 (in=x2);

by Tester2 GRP G;

if x1=**1** and x2=**1**;

**run**;

**Proc** **sort** data=KWS_2011_5_fold;

by Replicate _Block Tester2 GRP G;

**run**;

/*----USE file "D:\Joseph2013\Synbreed_2010_2013\Models"KWS_2010_2012_export" to export KWS_2011_5_fold*/;

/*--------------Split KWS 2012 dataset into 5 parts using stratified random sampling------*/;

**data** Geno_pheno_2012_1; set "D:\Joseph2013\Synbreed_2010_2013\Results\geno_pheno_2012" ;

rename labcode = G;

keep Tester2 GRP labcode Estimate ;

**run**;

**%macro** ***replicate_2012***;

%put START TIME 2: %sysfunc(datetime(),datetime20.);

%do r=**1** %to **10**;

%let seed=%eval(1200+100*&r);

%let probvector=0.20 0.20 0.2 0.2 0.2;

%let control_vars=Tester2 GRP;

%let in_dsn=Geno_pheno_2012_1;

%let out_dsn=Lsmeans_2012_5_fold_rep&r;

%let sort=NEST;

%***split***(&seed, &probvector, &control_vars, &in_dsn, out_dsn=&out_dsn);

*options nomprint nomlogic;

%put END TIME: %sysfunc(datetime(),datetime20.);

;;

data Lsmeans_2012_5_fold_rep&r; set Lsmeans_2012_5_fold_rep&r;

Replicate=&r;

run;

%end;

**%mend**;

%***replicate_2012***;

**data** Lsmeans_2012_5_fold_rep; set Lsmeans_2012_5_fold_rep:; **run**;

**Proc** **sort** data=Lsmeans_2012_5_fold_rep;

by Replicate _BLOCK;

**run**;

**proc** **freq** data=Lsmeans_2012_5_fold_rep noprint;

by replicate;

tables _BLOCK*Tester2*GRP /missing out=_freq_out_2012;

**run**;

**proc** **means** data=_freq_out_2012 noprint nway;

by Replicate;

class _BLOCK;

var count percent;

output out=summary_2012(drop=_type_ _freq_) sum=;

**run**;

**data** Geno_pheno_2012_2; set "D:\Joseph2013\Synbreed_2010_2013\Results\geno_pheno_2012" ;

rename labcode = G;

**run**;

**proc** **sort** data = Geno_pheno_2012_2;

by Tester2 GRP G;

**run**;

**proc** **sort** data=Lsmeans_2012_5_fold_rep;

by Tester2 GRP G;

**run**;

**data** KWS_2012_5_fold;

merge Lsmeans_2012_5_fold_rep (in=x1) Geno_pheno_2012_2 (in=x2);

by Tester2 GRP G;

if x1=**1** and x2=**1**;

**run**;

**Proc** **sort** data=KWS_2012_5_fold;

by Replicate _Block Tester2 GRP G;

**run**;

/*---------------------------Removing effect of Tester*GRP-------*/;

**Proc** **sort** data=KWS_2010_5_fold;

by Tester2 GRP G;

**run**;

**Proc** **sort** data="D:\Joseph2013\Synbreed_2010_2013\Results\tester_grp_effect_2010_2" out=tester_grp_effect_2010_2;

by Tester2 GRP;

**run**;

**data** KWS_2010_5_fold_b;

merge KWS_2010_5_fold tester_grp_effect_2010_2(where=(Tester2 in ('T1', 'T2') and GRP in ('G1','G2','G3')));

by Tester2 GRP;

Estimate=Estimate-Effect;

drop Effect;

**run**;

**Proc** **sort** data=KWS_2010_5_fold_b;

by Replicate _Block Tester2 GRP G;

**run**;

**Proc** **sort** data=KWS_2011_5_fold;

by Tester2 GRP G;

**run**;

**Proc** **sort** data="D:\Joseph2013\Synbreed_2010_2013\Results\tester_grp_effect_2011_2" out=tester_grp_effect_2011_2;

by Tester2 GRP;

**run**;

**data** KWS_2011_5_fold_b;

merge KWS_2011_5_fold tester_grp_effect_2011_2(where=(Tester2 in ('T1', 'T2') and GRP in ('G1','G2','G3')));

by Tester2 GRP;

Estimate=Estimate-Effect;

drop Effect;

**run**;

**Proc** **sort** data=KWS_2011_5_fold_b;

by Replicate _Block Tester2 GRP G;

**run**;

**Proc** **sort** data=KWS_2012_5_fold;

by Tester2 GRP G;

**run**;

**Proc** **sort** data="D:\Joseph2013\Synbreed_2010_2013\Results\tester_grp_effect_2012_2" out=tester_grp_effect_2012_2;

by Tester2 GRP;

**run**;

**data** KWS_2012_5_fold_b;

merge KWS_2012_5_fold tester_grp_effect_2012_2(where=(Tester2 in ('T1', 'T6', 'T9') and GRP in ('G1','G2','G3')));

by Tester2 GRP;

Estimate=Estimate-Effect;

drop Effect;

**run**;

**Proc** **sort** data=KWS_2012_5_fold_b;

by Replicate _Block Tester2 GRP G;

**run**;
