# Supplementary material for: Genomic prediction using machine learning: a comparison of the performance of regularized regression, ensemble, instance-based and deep learning methods on synthetic and empirical data
Source: BMC Genomics. 2024 Feb 7;25:152. doi: 10.1186/s12864-023-09933-x (PMC10848392; doi:10.1186/s12864-023-09933-x)
Supplement: Supplementary file 5 — Additional file 5. Includes the RR-BLUP model used to estimate variance components for the KWS real maize data (Section 1), the Noteworthy details of model fitting (Section 2) plus the additional Tables of results (Section 3). Table S1. List of R and Python packages used in this paper. Table S2. Prediction accuracy (PA) of the regularized, adaptive regularized and Bayesian regularized methods, computed as the Pearson correlation coefficient between the true breeding values (TBVs) and the predicted breeding values (PBVs), for the simulated dataset, where $T_1-T_3$T1-T3 refer to three quantitative milk traits. The choice of $\lambda$λ, where applicable, was based on the 10-fold CV. The mean squared and absolute prediction errors are also provided. Table S3. Prediction accuracy (PA) of the group regularized methods (mean and range values of PA across the different groupings), computed as the Pearson correlation coefficient between the true breeding values (TBVs) and the predicted breeding values (PBVs), for the simulated dataset, where $T_1-T_3$T1-T3 refer to three quantitative milk traits. Choice of [file 12864_2023_9933_MOESM5_ESM.pdf]

Genomic prediction using machine learning: A comparison of the performance of regularized regression, ensemble, instance-based and deep learning methods on synthetic and empirical data

Supplementary materials

Vanda M. Lourenço[1], Joseph O. Ogutu[2], Rui A.P. Rodrigues[1], Alexandra Posekany[3], and Hans-Peter Piepho[2]

[1]Center for Mathematics and Applications (NOVA Math), NOVA FCT and Department of Mathematics, Caparica, Portugal
[2]Institute of Crop Science, Biostatistics Unit, University of Hohenheim, Stuttgart, Germany
[3]Research Unit of Computational Statistics, Vienna University of Technology, Vienna, Austria

1

Ridge Regression (RR)-BLUP model used to estimate variance components for the KWS real maize data

The linear mixed effects model used for the phenotypic analysis of the KWS real maize dataset, which is described in greater detail in [14, 15], is

$$y_{ijklqmn} = \phi + \gamma_q + t_l + r_{kl} + b_{jkl} + \delta_m + \tau_n + Z_{1im}g_{1im} + Z_{2im}g_{2im} + e_{ijklmnq} \quad (1.1)$$

where

- $y_{ijklqmn}$ is the quantitative response (maize yield) of the i-th genotype in the j-th block nested within the k-th replicate in the l-th trial in the qth location and in the m-th group tested against the n-th tester

- ϕ is the general effect

- γ_q is the random effect of the q-th location, which is iid according to $N(0, \sigma_\gamma^2)$, σ_γ^2 is the location variance

- t_{lq} is the random effect of the l-th trial nested within the q-th location, which is iid according to $N(0, \sigma_t^2)$, where σ_t^2 is the trial variance

- r_{klq} is the random effect of the k-th replicate nested within the l-trial in the q-th location, which is iid according to $N(0, \sigma_r^2)$, where σ_r^2 is the replicate variance

- b_{jklq} is the random effect of the j-th block nested within the k-th replicate in the l-th trial in the q-th location, which is iid according to $N(0, \sigma_b^2)$, where σ_b^2 is the block variance

- δ_m is the fixed effect of the m-th group of checks, testers and genotypes

- τ_n is the fixed effect of the n-th tester

- $Z_{1im}g_{1im}$ refers to the random effect of the i-th genotyped line in the m-th group, coded as (Z1*TESTER*GRP*G1), where Z1 is the quantitative variable Z_{1im} (SWITCH1) in model (1.1), tester (TESTER), group (GRP) and genotypes (G1) are categorical variables

- $Z_{2im}g_{2im}$ refers to the random effect of the i-th non-genotyped line in the m-th group, coded as (Z2*TESTER*GRP*G2), where Z2 is the quantitative variable Z_{2im} (SWITCH2) in model (1.1), tester (TESTER), group (GRP) and genotypes (G2) are categorical variables

- $e_{ijklmqn}$ is the residual plot error, which is iid according to $N(0, \sigma_e^2)$, where σ_e^2 is the error variance

We further note that,

- Variable G1 has one unique level for each of the genotyped lines and assigns any one level of the genotyped lines to all the non-genotyped lines. Hence G1=G2 for all the genotyped lines.

- Variable G2 has one unique level for each genotyped line and one unique level for each non-genotyped line.

- SWITCH1 is a dummy variable equal to 1 for genotyped lines and 0 for non-genotyped lines.

- SWITCH2 is a dummy variable equal to 0 for genotyped lines and 1 for non-genotyped lines.

- G1(=GENB) and G2(=GENA), SWITCH1 and SWITCH2 are explained in greater detail in the supporting information of [15].

2

Noteworthy details of model fitting

Because we used CV for model selection, we fixed the data split whenever possible by setting a specific positive seed before splitting the data to enhance the reproducibility of results. Besides the k-fold split, common to all the methods, some methods involve additional kinds of randomization, making it impossible to reproduce results even if the data split or the seed for the random number generator are fixed prior to fitting the model (RF, SVM and FFNN methods). This happens because some of the routines internally generate some random number seeds when they initialize the computations. Consequently, this introduces an additional source of variation between results obtained from different runs or even from using different computing platforms. Ideally, for such cases, and at the risk of vastly increasing the computational burden, the model could be fitted a large number of times until the average and the range (or standard error) of PA stabilize. Even so, we considered only single model runs for the RF and SVM methods. For the FFNN method we implemented 1000 runs for the simulated data and 10 for the KWS data.

In addition, the following details of model fitting are noteworthy:

(i) $\gamma = 0.5$ was considered in the bridge and grouped bridge methods.

(ii) Calibration of random forests involves selecting the number of trees to grow (n_{trees}), the random number of covariates to select for growing each tree (n_{covars}) and the minimum size of the terminal nodes per tree (n_{nodes}), below which no split is attempted. The parameters that affect the final accuracy the most are the first two. Increasing the n_{trees} only increases the accuracy up to some point but can substantially increase the computational time. Here, we fix $n_{trees} = 1000$ (this ensures that every input row gets predicted at least a couple of times) and $n_{nodes} = 1$ and search for the best value of n_{covars} in $\{0.5, 1, 2\} \times (p/3)$.

(iii) For the SGB method we assumed the Gaussian distribution for minimizing squared-error loss. The basic boosting algorithm requires the specification of two parameters: the number of splits (J; or the number of nodes, which equals the number of splits plus one) and the number of trees (or iterations; n_{trees}) to be fitted. Hastie et al [25] point out that the number of splits J such that $4 \leq J \leq 8$ generally works well with results being fairly insensitive to particular choices in this range. We use $J = 6$. As for n_{trees}, it should neither be too small (bad fit) nor too large (overfit). Usually, the suitable n_{trees} can be found by checking how well the model fits a test dataset, where a typical fraction of the data used for testing is 0.5 (can be much smaller if the dataset is very large). We search for n_{trees} in $\{500, 1500, 3000\}$. As with RF, we fix the number of terminal nodes per tree $n_{nodes} = 1$. In addition, we set the shrinkage factor applied to each tree in the expansion to 0.001 and the subsampling fraction to 0.5 [19].

(iv) For the SVR method, we considered an insensititvity zone of $\varepsilon = 0.001$ across traits with the regularization parameter λ (cost) determined by grid-search over the values $\{0.01, 0.1, 1, 10, 100\}$.

(v) We used the Adam optimizer, 'Relu' (rectified linear units) activation function and a linear output layer in the configuration of both the FFNN and CNN.

Because we used the Python software and GPU to fit the neural networks, we were able to produce 1000 different runs of the FFNNs and CNNs for the simulated dataset and 10 runs of the FFNNs for each of the three real datasets (amounting to 10 $runs \times 5\ folds \times 10\ reps$ FFNNs fits for each real dataset), and report the average plus the range for the PA.

(vi) For the 1D CNN we fixed the following parameters across the three traits:

**CNN**	$\mathbf{T_1/T_2/T_3}$
Number or filters (convolutional layer)	64
Filter size (convolutional layer)	36
Stride (convolutional layer)	1
Number of dense layers	3
Number of units (dense layer)	128
Number of maxpooling layers	1
Pooling size (maxpooling layer)	36
Stride (maxpooling layer)	18

Parameters referring to the Number of epochs and the Learning rate were calibrated individually for each trait (Table S7).

(vii) For the simulated animal trait T3, specifically with the CNN method, we applied the transformation $T3 \to T3 \times 10^3$. This was necessary because the CNN did not generate reasonable results with the original trait values, which are exceptionally small and close to zero. Subsequently, the prediction errors were back-transformed to the original scale and reported accordingly.

All the methods are implemented in the R software and are available in various R packages. For the FFNN and CNN methods, and because of fine tuning requirements, we used the `Python` software. Table S1 lists the `R and Python` packages we used to analyse the synthetic and real datasets.

3
Supplementary Tables

Table S1: List of R and Python packages used in this paper

Type	Method	Software/Package	Routine
**Regularized**	Bridge	R/grpreg [8]	cv.grpreg()
	RR-CV, ENET	R/glmnet [21]	cv.glmnet() *
	RR-REML	R/rrBLUP [12]	mixed.solve()
	LASSO, SCAD, MCP	R/ncvreg [9]	cv.ncvreg() *
**Sparse regularized**	ENET	R/sparsenet [38]	cv.sparsenet()
**Adaptive regularized**	LASSO	R/glmnet [21]	cv.glmnet() *
	SCAD		asnet()*
	MCP	R/msaenet [65]	amnet()*
	ENET		aenet()*, msaenet()*
**Group regularized**	Hierarchical LASSO	R/glinternet [37]	glinternet.cv()
	Sparse LASSO	R/SGL [61]	cvSGL()
	Bridge		
	LASSO, MCP, SCAD	R/grpreg [8]	cv.grpreg()
	gel, cMCP (bi-level)		
**Bayesian regularized**	rBayes, lBayes	R/BGLR [52]	BGLR()
**Ensemble**	Random Forests	R/randomForest [10]	randomForest()
	Stochastic Gradient Boosting	R/gbm [24]	gbm.fit()
**Instance-based**	Support Vector Machine	R/e1071 [40]	tune.svm()
**Deep learning**	Feed-Forward Neural Network	Python/Pandas [39]	-
	Convolutional Neural Network	Python/TensorFlow [1]	-
		Python/Numpy [39]	-

* These functions allow for internal parallelization of computations.

Table S2: Prediction accuracy (PA) of the **regularized**, **adaptive regularized** and **Bayesian regularized** methods, computed as the Pearson correlation coefficient between the true breeding values (TBVs) and the predicted breeding values (PBVs), for the simulated dataset, where $T_1 - T_3$ refer to three quantitative milk traits. The choice of λ, where applicable, was based on the 10-fold CV. The mean squared and absolute prediction errors are also provided.

**Method**	$\mathbf{T_1}$			$\mathbf{T_2}$			$\mathbf{T_3}^\star$		
	PA	MSPE	MAPE	PA	MSPE	MAPE	PA	MSPE	MAPE
bridge	0.716	7133.7	68.1	0.751	47.2	5.9	0.769	0.922	0.283
RR-CV	0.736	6561.0	64.9	0.771	52.2	6.3	0.762	0.928	0.283
RR-REML	0.732	6570.3	65.0	0.770	51.4	6.2	0.758	0.931	0.283
LASSO	0.775	5951.3	61.7	0.827	47.3	6.1	0.817	0.920	0.286
ENET	0.777	5957.6	61.7	0.829	47.2	6.0	0.817	0.921	0.286
sENET	0.779	5920.2	61.5	0.814	47.8	6.1	0.817	0.914	0.285
SCAD	0.775	5878.8	61.1	0.815	48.2	6.1	0.808	0.922	0.286
MCP	0.742	6308.4	63.1	0.790	49.9	6.2	0.799	0.921	0.285
aLASSO	0.724	6591.5	65.8	0.779	48.3	6.0	0.796	0.966	0.293
aENET^e	0.726	6812.1	65.1	0.786	49.6	6.1	0.805	0.915	0.285
aENET^r	0.722	6674.6	66.0	0.789	47.3	6.0	0.801	0.950	0.290
maENET^e	0.704	7231.0	67.1	0.773	50.7	6.2	0.791	0.915	0.283
maENET^r	0.692	7117.4	68.2	0.756	52.4	6.3	0.767	0.892	0.278
aSCAD^s	0.645	7732.4	70.8	0.739	56.6	6.5	0.750	0.926	0.282
aSCAD^r	0.700	6833.5	66.3	0.721	58.5	6.7	0.730	0.892	0.275
aMCP^m	0.682	7323.4	68.8	0.754	56.4	6.5	0.755	0.922	0.282
aMCP^r	0.681	6987.3	66.9	0.714	59.2	6.7	0.734	0.884	0.274
rBayes	0.730	6606.4	65.0	0.767	51.4	6.2	0.756	0.932	0.284
lBayes	0.763	6141.2	62.7	0.807	48.1	6.1	0.794	0.930	0.286

s: sparse method; a: adaptive method; ma: multi-step adaptive method; r: ridge method; l: lasso method.

s: *snet* penalty; *r*: *ridge* penalty; *m*: *mnet* penalty; *e*: *enet* penalty;

* MSPE is multiplied by 10^3 and MAPE by 10^1 to enhance comparison with corresponding values for T_1 and T_2.

Table S3: Prediction accuracy (PA) of the **group regularized** methods (mean and range values of PA across the different groupings), computed as the Pearson correlation coefficient between the true breeding values (TBVs) and the predicted breeding values (PBVs), for the simulated dataset, where $T_1 - T_3$ refer to three quantitative milk traits. Choice of λ was based on the 10-fold CV. Display refers to the mean, max and min values of PA across all the 10 grouping schemes. The mean squared and absolute prediction errors are also provided.

**Method**		$\mathbf{T_1}$		$\mathbf{T_2}$		$\mathbf{T_3}^{\star}$	
		Mean	Range	Mean	Range	Mean	Range
gbridge	PA	0.716	0.659–0.762	0.813	0.794–0.829	0.803	0.779-0.816
	MSPE	6468.7	5957.5–7095.8	48.9	44.5–52.5	0.919	0.907–0.946
	MAPE	64.6	62.0–67.4	6.1	5.8–6.4	0.285	0.284–0.288
gLASSO	PA	0.766	0.732–0.793	0.820	0.795–0.852	0.814	0.802–0.838
	MSPE	6047.3	5484.3–6533.3	49.9	47.7–52.4	0.902	0.881–0.924
	MAPE	62.3	59.6–64.4	6.2	6.1–6.3	0.282	0.277–0.288
sgLASSO	PA	0.724	0.722–0.724	0.792	0.790–0.804	0.809	0.784–0.815
	MSPE	7392.1	7378.1–7420.9	47.9	47.6–48.2	0.951	0.929–0.957
	MAPE	68.5	68.4–68.7	6.0	6.0–6.1	0.291	0.284–0.292
gSCAD	PA	0.763	0.731–0.793	0.820	0.799–0.843	0.807	0.789–0.822
	MSPE	6059.5	5314.7–6595.2	48.4	46.9–51.1	0.901	0.862–0.929
	MAPE	62.4	58.8–64.7	6.1	6.0–6.3	0.282	0.273–0.288
gMCP	PA	0.742	0.701–0.776	0.806	0.775–0.832	0.799	0.779–0.821
	MSPE	6239.8	5273.2–7353.6	48.6	46.6–50.8	0.899	0.859–0.951
	MAPE	63.5	58.9–69.1	6.1	6.0–6.3	0.281	0.274–0.290
cMCP	PA	0.653	0.633–0.655	0.808	0.808–0.809	0.8176	0.8175–0.8177
	MSPE	7430.9	7368.8–7989.9	48.5	48.4–48.6	0.91935	0.91930–0.91943
	MAPE	68.6	68.4–70.6	6.11	6.10–6.12	0.28607	0.28606–0.28611
gel	PA	0.677	0.623–0.756	0.758	0.697–0.800	0.765	0.744–0.804
	MSPE	7732.3	5839.5–9099.4	51.0	48.2–53.0	0.915	0.873–0.970
	MAPE	70.4	60.8–77.0	6.2	6.0–6.4	0.281	0.273–0.290
hLASSO*	PA	0.755	-	0.819	-	0.795	-
	MSPE	6592.6	-	49.9	-	0.901	-
	MAPE	65.5	-	6.2	-	0.280	-

s and h stand for the sparse and hierarchical versions of the corresponding method;

* the groupings here are achieved through interactions between markers unlike in the previous methods; a single PA value is produced and reported, which is not a mean across the different groups;

* MSPE is multiplied by 10^3 and MAPE by 10^1 to enhance comparison with corresponding values for T_1 and T_2; five decimal places were needed for cMCP.

Table S4: Prediction accuracy (PA) of the **ensemble and instance-based** methods, computed as the Pearson correlation coefficient between the true breeding values (TBVs) and the predicted breeding values (PBVs), for the simulated dataset, where $T_1 - T_3$ refer to three quantitative milk traits.

**Method**	$\mathbf{T_1}$			$\mathbf{T_2}$			$\mathbf{T_3}^{\star}$		
	PA	MSPE	MAPE	PA	MSPE	MAPE	PA	MSPE	MAPE
Random Forests (RF)†	0.741	7945.8	72.4	0.788	58.6	6.5	0.713	0.924	0.272
Stochastic Gradient Boosting (SGB)*	0.690	8382.5	73.8	0.725	67.6	7.0	0.676	0.950	0.275
Support Vector Machines (SVM)‡	0.695	7102.1	67.2	0.740	53.9	6.4	0.731	0.934	0.282

† reported values refer to a single run of the random forest with the subset of markers selected randomly for growing each tree set equal to $0.5 \times \frac{p}{3}$;

* the best number of trees grown was 3000 for all traits;

‡ reported values refer to a single run of the SVM with the best cost $\lambda = 100$ for trait 1 and $\lambda = 10$ for traits 2 and 3;

⋆ MSPE is multiplied by 10^3 and MAPE by 10^1 to enhance comparison with corresponding values for T_1 and T_2.

Table S5: Prediction accuracy (PA) of the **deep learning** methods, computed as the Pearson correlation coefficient between the true breeding values (TBVs) and the predicted breeding values (PBVs), for the simulated dataset, where $T_1 - T_3$ refer to three quantitative milk traits.

		$\mathbf{T_1}$		$\mathbf{T_2}$		$\mathbf{T_3}^{\star}$	
		Mean	Range	Mean	Range	Mean	Range
Feed-Forward Neural Networks (FFNN)†	PA	0.705	0.692–0.717	0.725	0.705–0.741	0.703	0.651–0.731
	MSPE	8092.5	6046.5–13556.7	60.6	32.5–102.4	0.974	0.464–1.790
	MAPE	72.1	61.6–95.4	6.6	4.6–9.1	0.284	0.183–0.402
Convolutional Neural Networks (CNN)†	PA	0.601	0.537–0.668	0.665	0.596–0.702	0.649	0.495–0.683
	MSPE	13129.2	7996.1–22305.3	56.7	0.8–96.2	0.957	0.849–1.229
	MAPE	92.0	72.0–128.2	6.4	4.5–8.8	0.278	0.260–0.318

† values refer to 1000 different runs of the neural network;

⋆ MSPE is multiplied by 10^3 and MAPE by 10^1 to enhance comparison between methods.

Table S6: Best FFNN model calibration parameters selected for each of the three quantitative milk traits $T_1 - T_3$.

**FFNN**	$\mathbf{T_1}$	$\mathbf{T_2}$	$\mathbf{T_3}$
Number of hidden layers	1	3	3
Number of units (hidden layer)	200	800	400
Number of epochs	200	260	300
Dropout rate (input layer)	0.1	0.15	0.15
Batch size	128	32	16
Learning rate	10^{-4}	2×10^{-5}	2×10^{-5}
Dropout rate (hidden layers)	0.8	0.8	0.5
Dropout rate (last hidden layer)	0.8	0.88	0.775
Batch normalization (hidden layer)	No	Yes	Yes

Table S7: Best CNN model calibration parameters (Number of epochs/Learning rate) selected for each of the three quantitative milk traits $T_1 - T_3$.

**Steps**	$\mathbf{T_1}$	$\mathbf{T_2}$	$\mathbf{T_3}$
1	$50/10^{-3}$	$30/10^{-3}$	$50/10^{-3}$
2	$50/10^{-4}$	$10/10^{-4}$	$100/10^{-4}$
3	-	-	$500/10^{-5}$

Table S8: Predictive ability (PA; mean and range values computed across the 5-fold validation datasets and 10 replicates) of the regularized, adaptive regularized, group regularized, Bayesian regularized, ensemble, instance-based and deep learning methods, computed as the Pearson correlation coefficient between the observed breeding values (OBVs) and the predicted breeding values (PBVs), for the KWS datasets. The choice of λ, where applicable, was based on 4-fold CV.

**Method**		**2010**		**2011**		**2012**	
		Mean	Range	Mean	Range	Mean	Range
RR-CV	PA	0.632	0.529-0.724	0.555	0.441-0.648	0.598	0.549-0.674
	MSPE	41.9	31.7-58.3	46.4	35.5-62.1	34.5	29.1-41.5
	MAPE	4.7	4.1-5.2	5.2	4.6-6.1	4.6	4.3-5.0
RR-REML	PA	0.649	0.523-0.725	0.576	0.469-0.663	0.616	0.555-0.678
	MSPE	41.2	31.2-59.2	44.4	35.4-63.4	33.4	27.8-40.2
	MAPE	4.6	4.0-5.2	5.0	4.5-6.0	4.5	4.1-4.9
sENET	PA	0.626	0.502-0.723	0.527	0.372-0.639	0.596	0.531–0.697
	MSPE	42.4	32.6–62.4	48.8	37.2–84.5	34.7	28.0–42.2
	MAPE	4.7	4.3–5.3	5.3	4.6–6.8	4.6	4.2–5.1
aENET^e	PA	0.592	0.453-0.701	0.482	0.326-0.651	0.572	0.501-0.676
	MSPE	46.5	37.4-67.6	54.3	37.7-98.9	37.0	30.6-46.3
	MAPE	4.9	4.5-5.6	5.6	4.9-7.2	4.7	4.4-5.1
gLASSO‡	PA	0.584	0.494–0.673	0.512	0.404–0.606	0.586	0.514–0.679
	MSPE	46.3	36.0–59.8	49.5	37.5–65.6	35.5	28.1–42.5
	MAPE	4.9	4.3–5.5	5.4	4.6–6.3	4.7	4.2–5.1
Random Forests†	PA	0.656	0.549–0.727	0.570	0.474–0.659	0.556	0.484–0.633
	MSPE	39.7	28.9–56.3	45.7	34.1–61.4	37.3	29.9–46.3
	MAPE	4.6	4.0–5.1	5.1	4.4–6.0	4.8	4.3–5.3
lBayes	PA	0.649	0.522–0.723	0.587	0.467–0.658	0.624	0.556–0.679
	MSPE	40.2	31.6–59.5	46.4	37.7–62.3	31.7	27.8–40.2
	MAPE	4.6	4.0–5.2	5.0	4.6–6.0	4.4	4.1–4.9
Feed-Forward Neural Networks*	PA	0.634	0.514–0.728	0.541	0.412–0.656	0.586	0.512–0.663
	MSPE	42.9	31.9–61.7	49.6	38.4 –75.1	36.5	29.0–48.5
	MAPE	4.7	4.0–5.4	5.3	4.6 –6.6	4.7	4.2–5.3

a: adaptive method; s: sparse method; g: grouped method; l: lasso method; *e*: *enet* penalty;

‡reported values refer to the grouping indexes (or sizes) 50, 30 and 80 for the 2010, 2011 & 2012 datasets, respectively;

†reported values refer to a single run of the random forest with the subset of markers selected randomly for growing each tree set equal to $0.5 \times \frac{p}{3}$ for the 2010 and 2011 datasets but to $2 \times \frac{p}{3}$ for the 2012 dataset;

* The best FFNN performing model, in terms of PA, for the 2010 and 2011 datasets was the one used for trait T_3 from the simulated data, whereas for the 2012 dataset, it was the one used for trait T_1 from the simulated data.

Bibliography

[1] TensorFlow Eager: A multi-stage, Python-embedded DSL for machine learning. Proceedings of Machine Learning and Systems, 1, 178–189.

[2] Bach, F. (2008). Consistency of the group lasso and multiple kernel learning. Journal of Machine Learning, 9, 1179–1225.

[3] Bengio, Y. (2012). Practical recommendations for gradient-based training of deep architectures. In Neural Networks: Tricks of the trade, 437–478. Springer, Berlin, Heidelberg.

[4] Bien, J., Taylor, J. & Tibshirani, R. (2013). A lasso for hierarchical interactions. The Annals of Statistics, 41, 1111–1141.

[5] Breheny, P. & Huang, J. (2009). Penalized methods for bi-level variable selection. Statistics Interface, 2, 369–380.

[6] Breheny, P. & Huang, J. (2011). Coordinate descent algorithms for nonconvex penalized regression, with applications to biological feature selection. Annals of Applied Statistics, 5, 232–253.

[7] Breheny, P., & Huang, J. (2015). Group descent algorithms for nonconvex penalized linear and logistic regression models with grouped predictors. Statistics and Computing, 25(2), 173–187.

[8] Breheny, P. & Breheny, M. P. (2021). Package 'grpreg'.

[9] Breheny, P. & Breheny, M. P. (2021). Package 'ncvreg'.

[10] Breiman, L. (2001). Random forests. Machine Learning, 45, 5–32.

[11] Chen, Z., Zhu, Y. & Zhu, C. (2016). Adaptive bridge estimation for high-dimensional regression models. Journal of Inequalities and Applications, 1, 258.

[12] Endelman, J. B. (2011). Ridge regression and other kernels for genomic selection with R package rrBLUP. The plant genome, 4(3).

[13] Eraslan, G., Avsec, Ž., Gagneur, J. & Theis, F.J. (2019). Deep learning: new computational modelling techniques for genomics. Nature Reviews Genetics, 20(7), 389–403.

[14] Estaghvirou, S. B. O., Ogutu, J. O., Schulz-Streeck, T., Knaak, C., Ouzunova, M., Gordillo, A., & Piepho, H. P. (2013). Evaluation of approaches for estimating the accuracy of genomic prediction in plant breeding. BMC Genomics, 14(1), 1–21.

[15] Estaghvirou, S. B. O., Ogutu, J. O., & Piepho, H. P. (2015). How genetic variance and number of genotypes and markers influence estimates of genomic prediction accuracy in plant breeding. Crop Science, 55(5), 1911–1924.

[16] Fan, J. & Li, R. (2001). Variable selection via nonconcave penalized likelihood and its oracle properties. Journal of the American Statistical Association, 96, 1348–1360.

[17] Fan, J. & Peng, H. (2004). Nonconcave penalized likelihood with a diverging number of parameters. Annals of Statistics, 32, 928–961.

[18] Frank, I.E. & Friedman, J.H. (1993). A statistical view of some chemometrics regression tools (with discussion). Technometrics, 35, 109-148.

[19] Friedman, J. (2001). Greedy function approximation: a gradient boosting machine. Annals of Statistics, 29, 1189–1232.

[20] Friedman, J., Hastie, T. & Tibshirani, R. (2010). A note on the group lasso and sparse group lasso. arXiv preprint arXiv:1001.0736.

[21] Friedman, J., Hastie, T., Tibshirani, R., Narasimhan, B., Tay, K., Simon, N., & Qian, J. (2022). Package 'glmnet'. Journal of Statistical Software. 2010a, 33(1).

[22] Fu, W.J. (1998). Penalized regressions: The bridge versus the lasso. Journal of Computational and Graphical Statistics, 7, 397–416.

[23] Grandvalet, Y. (1998). Least absolute shrinkage is equivalent to quadratic penalization. International Conference on Artificial Neural Networks, 201–206). Springer, London.

[24] Greenwell, B., Boehmke, B., Cunningham, J., Developers, G. B. M. & Greenwell, M. B. (2019). Package 'gbm'.

[25] Hastie, T.J., Tibshirani, R. & Friedman, J. (2009). The elements of statistical learning, Second edition, New York: Springer.

[26] Hayes, B. J., Visscher, P. M. & Goddard, M. E. (2009). Increased accuracy of artificial selection by using the realized relationship matrix. Genetics Research, 91(1), 47–60.

[27] Heslot, N., Yang, H.P., Sorrells, M.E. & Jannink, J.L. (2012). Genomic selection in plant breeding: a comparison of models. Crop Science, 52, 146–160.

[28] Hoerl, A.E. & Kennard, R.W. (1970). Ridge regression: biased estimation for non-orthogonal problems. Technometrics, 12, 55–67.

[29] Huang, J., Ma, S., Xie, H. & Zhang, C-H. (2009). A group bridge approach for variable selection. Biometrika, 96, 339–355.

[30] Huang, J., Horowitz, J.L. & Ma, S. (2008). Asymptotic properties of bridge estimators in sparse high-dimensional regression models. Annals of Statistics, 36, 587–613.

[31] Huang, J. & Zhang, T. (2010). The benefit of group sparsity. Annals of Statistics, 38, 1978–2004.

[32] Huang, J., Breheny, P. & Ma, S. (2012). A Selective Review of Group Selection in High-Dimensional Models. Statistical Science, 27(4), 10.1214/12-STS392.

[33] Kim, Y., Choi, H. & Oh, H. S. (2008). Smoothly clipped absolute deviation on high dimensions. Journal of the American Statistical Association, 103(484), 1665–1673.

[34] Kingma, D.P. & Ba, J.L. (2014). Adam: A method for stochastic optimization. arXiv preprint arXiv:1412.6980.

[35] Knight, K. & Fu, W. (2000). Asymptotics for Lasso-type estimators. Annals of Statistics, 28, 356–1378.

[36] Liaw, A. & Wiener, M. (2002). Classification and regression by randomForest. R News, 2, 18–22.

[37] Lim, M. & Hastie, T. (2015). Learning interactions via hierarchical group-lasso regularization. Journal of Computational and Graphical Statistics, 24(3), 627–654.

[38] Mazumder, R., Friedman, J.H. & Hastie, T. (2011). Sparsenet: Coordinate descent with nonconvex penalties. Journal of the American Statistical Association, 106(495), 1125–1138.

[39] McKinney, W. (2012). Python for data analysis: Data wrangling with Pandas, NumPy, and IPython. O'Reilly Media, Inc.

[40] Meyer, D., Dimitriadou, E., Hornik, K., Weingessel, A., Leisch, F., Chang, C. C. et al (2019). Package 'e1071'. The R Journal.

[41] Meuwissen, T. H., Hayes, B. J. & Goddard, M. (2001). Prediction of total genetic value using genome-wide dense marker maps. Genetics, 157(4), 1819–1829.

[42] Min, S., Lee, B. & Yoon, S. (2017). Deep learning in bioinformatics. Briefings in Bioinformatics, 18(5), 851–869.

[43] Montesinos-López, A., Montesinos-López, O.A., Gianola, D., Crossa, J. & Hernández-Suárez, C.M. (2018). Multi-environment genomic prediction of plant traits using deep learners with dense architecture. G3: Genes, Genomes, Genetics, 8(12), 3813–3828.

[44] Montesinos-López, O.A., Montesinos-López, A., Crossa, J., Gianola, D., Hernández-Suárez, C.M., & Martín-Vallejo, J. (2018). Multi-trait, multi-environment deep learning modeling for genomic-enabled prediction of plant traits. G3: Genes, Genomes, Genetics, 8(12), 3829–3840.

[45] Montesinos-López, O.A., Martín-Vallejo, J., Crossa, J., Gianola, D., Hernández-Suárez, C.M., Montesinos-López, A., Philomin J. & Singh, R. (2019). A benchmarking between deep learning, support vector machine and Bayesian threshold best linear unbiased prediction for predicting ordinal traits in plant breeding. G3: Genes, Genomes, Genetics, 9(2), 601–618.

[46] Montesinos-López, O.A., Martín-Vallejo, J., Crossa, J., Gianola, D., Hernández-Suárez, C.M., Montesinos-López, A., Juliana, P. & Singh, R., (2019). New deep learning genomic-based prediction model for multiple traits with binary, ordinal, and continuous phenotypes. G3: Genes, Genomes, Genetics, 9(5), 1545–1556.

[47] Ogutu, J.O., Piepho, H-P. & Schultz-Streeck, T. (2011). A comparison of random forests, boosting and support vector machines for genomic selection. BMC Proceedings, 5(3), BioMed Central Ltd.

[48] Ogutu, J.O., Schulz-Streeck, T. & Piepho H-P. (2012). Genomic selection using regularized linear regression models: ridge regression, lasso, elastic net and their extensions. BMC Proceedings, 6(2), BioMed Central Ltd.

[49] Ogutu, J.O. & Piepho, H-P. (2014). Regularized group regression methods for genomic prediction: Bridge, MCP, SCAD, group bridge, group lasso, sparse group lasso, group MCP and group SCAD. BMC Proceedings, 8(5), BioMed Central Ltd.

[50] Park, C. & Yoon, Y.J. (2011). Bridge regression: adaptivity and group selection. Journal of Statistical Planning and Inference, 141, 3506–3519.

[51] Percival, D. (2011). Theoretical properties of the overlapping groups lasso. Electronic Journal of Statistics, 6, 269–288.

[52] Pérez, P. & de los Campos, G. (2014). Genome-wide regression and prediction with the BGLR statistical package. Genetics, 198, 483–495.

[53] Pérez-Enciso, M. & Zingaretti, L.M. (2019). A Guide on Deep Learning for Complex Trait Genomic Prediction. Genes, 10(7), p.553.

[54] Piepho H-P. (2009). Ridge regression and extensions for genomewide selection in maize. Crop Science, 49, 1165–1176.

[55] Piepho, H-P., Ogutu, J.O., Schulz-Streeck, T., Estaghvirou, B., Gordillo, A. & Technow, F. (2012). Efficient computation of ridge-regression best linear unbiased prediction in genomic selection in plant breeding. Crop Science, 52, 1093–1104.

[56] Poignard, B. (2020). Asymptotic theory of the adaptive Sparse Group Lasso. Annals of the Institute of Statistical Mathematics, 72(1), 297–328.

[57] Ruder, S. (2016). An overview of gradient descent optimization algorithms. arXiv preprint arXiv:1609.04747.

[58] Ruppert, D., Wand, M. P., & Carroll, R. J. (2003). Semiparametric regression. Cambridge University Press.

[59] Schonlau, M. (2005). Boosted regression (boosting): An introductory tutorial and a Stata plugin. The Stata Journal, 5(3), 330–354.

[60] Searle S. R., Casella G., McCulloch C. E. (1992). Variance components. Wiley, New York

[61] Simon, N., Friedman, J., Hastie, T. & Tibshirani, R. (2013). A sparse-group lasso. Journal of Computational and Graphical Statistics, 22, 231–245.

[62] Tibshirani, R. (1996). Regression shrinkage and selection via the lasso. Journal of the Royal Statistical Society, Series B, 58, 267–288.

[63] Usai, M. G., Gaspa, G., Macciotta, N. P., Carta, A. & Casu, S. (2014). XVIth QTLMAS: simulated dataset and comparative analysis of submitted results for QTL mapping and genomic evaluation. In: BMC proceedings. BioMed Central, 8(5), 1–9.

[64] Vapnik, V. (1995). The Nature of Statistical Learning Theory. Springer, New York.

[65] Xiao, N. & Xu, Q. S. (2015). Multi-step adaptive elastic-net: reducing false positives in high-dimensional variable selection. Journal of Statistical Computation and Simulation, 85(18), 3755–3765.

[66] Xie, L. (2009). Randomly split SAS data set exactly according to a given probability Vector.
https://silo.tips/download/randomly-split-sas-data-set-exactly-according-to-a-given-probability-vector

[67] Yuan, M. & Lin, Y. (2006). Model selection and estimation in regression with grouped variables. Journal of the Royal Statistical Society, Series B, 68, 49–67.

[68] Yue, T. & Wang, H. (2018). Deep learning for genomics: A concise overview. arXiv preprint arXiv:1802.00810.

[69] Zhang, C-H. (2007). Penalized linear unbiased selection. Department of Statistics and Bioinformatics, Rutgers University, Technical Report #2007-003.

[70] Zhang, C-H. & Huang, J. (2008.) The sparsity and bias of the lasso selection in high-dimensional linear regression. The Annals of Statistics, 36, 1567–1594.

[71] Zhang, C-H. (2010). Nearly unbiased variable selection under minimax concave penalty. Annals of Statistics, 38, 894–942.

[72] Zhou, N. & Zhu, J. (2010). Group variable selection via a hierarchical lasso and its oracle property. Statistics and its Interface, 3, 557–574.

[73] Zou, H. & Hastie, T. (2005). Regularization and variable selection via the elastic net. Journal of the Royal Statistical Association, Series B, 67, 301–320.

[74] Zou, H. (2006). The adaptive lasso and its oracle properties. Journal of the American Statistical Association, 101, 1418–1429.

[75] Zou, H,. Hastie, T. & Tibshirani, R. (2006). Sparse principal component analysis. Journal of Computational and Graphical Statistics, 15(2), 265–286.

[76] Zou, H. & Zhang, H.H. (2009). On the adaptive elastic-net with a diverging number of parameters. The Annals of Statistics, 37(4), 1733–1751.

[77] Zou, J., Huss, M., Abid, A., Mohammadi, P., Torkamani, A. & Telenti, A. (2019). A primer on deep learning in genomics. Nature Genetics, 51(1), 12–18.
